# Deficiency in peptidoglycan recycling promotes β-lactam sensitivity in *Caulobacter crescentus*

**DOI:** 10.1128/mbio.02975-24

**Published:** 2025-03-11

**Authors:** Malvika Modi, Deepika Chauhan, Michael C. Gilmore, Felipe Cava, Richa Priyadarshini

**Affiliations:** 1 Department of Life Sciences, School of Natural Sciences, Shiv Nadar Institution of Eminence 310513 , Gautam Buddha Nagar, Uttar Pradesh, India; 2 Laboratory for Molecular Infection Medicine Sweden, Department of Molecular Biology, Umeå Centre for Microbial Research, Umeå University 8075 , Umeå, Sweden; Fred Hutchinson Cancer Center, Seattle, Washington, USA; University of Wisconsin—Madison, Madison, Wisconsin, USA

**Keywords:** cell wall, β-lactam, peptidoglycan recycling, *Caulobacter crescentus*, antibiotic resistance

## Abstract

Peptidoglycan (PG)-modifying enzymes play a crucial role in cell wall remodeling, essential for growth and division. Cell wall degradation products are transported to the cytoplasm and recycled back in most gram-negative bacteria, and PG recycling is also linked to β-lactam resistance in many bacteria. *Caulobacter crescentus* is intrinsically resistant to β-lactams. Recently, it was shown that a soluble lytic transglycosylase, SdpA, is essential for β-lactam resistance. However, the precise role of SdpA in β-lactam resistance is unknown. This study investigated the PG recycling pathway and its role in antibiotic resistance in *C. crescentus*. Anhydromuropeptides generated by the action of lytic transglycosylases (LTs) are transported to the cytoplasm by the permease AmpG. *C. crescentus* encodes an *ampG* homolog, and deletion mutants of *sdpA* and *ampG* are sensitive to β-lactams. The *ampG* deletion mutant displays a significant accumulation of anhydromuropeptides in the periplasm of *C. crescentus,* demonstrating its essential role in PG recycling. While single knockout mutants of *sdpA* and *ampG* exhibit no growth defects, double-deletion mutants (∆*sdpA*∆*ampG*) exhibit severe growth and morphological defects. These double mutants also show enhanced sensitivity to β-lactams. Analysis of soluble muropeptides in wild-type (WT), ∆*sdpA*, and ∆*ampG* mutants revealed reduced levels of PG precursors (UDP-GlcNAc, UDP-MurNAc, and UDP-MurNAc-P5), suggesting that PG recycling products contribute toward *de novo* PG biosynthesis. Furthermore, supplementing the growth media with GlcNAc sugar enhanced the fitness of ∆*sdpA* and ∆*ampG* mutants under β-lactam stress. In conclusion, our study indicates that defects in PG recycling compromise cell wall biogenesis, leading to antibiotic sensitivity in *C. crescentus*.

**IMPORTANCE:**

β-lactam antibiotics target the peptidoglycan cell wall biosynthetic pathway in bacteria. In response to antibiotic pressures, bacteria have developed various resistance mechanisms. In many gram-negative species, cell wall degradation products are transported into the cytoplasm and induce the expression of β-lactamase enzymes. In this study, we investigated the cell wall recycling pathway and its role in antibiotic resistance in *Caulobacter crescentus*. Based on our data and prior studies, we propose that cell wall degradation products are utilized for the synthesis of peptidoglycan precursors in the cytoplasm. A deficiency in cell wall recycling leads to cell wall defects and increased antibiotic sensitivity in *C. crescentus*. These findings are crucial for understanding antibiotic resistance mechanisms in bacteria.

## INTRODUCTION

Besides providing cell shape, the cell wall also provides mechanical strength to withstand changes in the osmotic pressure (1, 2). The chemical structure of *N*-acetylglucosamine-*N*-acetylmuramic acid-pentapeptide (GlcNAc-MurNAc-P5) is found in almost all gram-negative and some gram-positive bacteria like *Bacillus subtilis* (3). The cell wall is constantly remodeled to accommodate cell growth and division (4). This complex task is achieved by a highly coordinated multienzyme complex composed of both cell wall degrading and synthesizing enzymes (2, 5).

The peptidoglycan (PG) layer must undergo cleavage to insert new PG subunits (6). PG-remodeling enzymes catalyze this PG cleavage. This group comprises the following. (i) *N*-acetylmuramoyl-L-alanine amidases break the amide bond between MurNAc and the stem peptide (7, 8). (ii) Muramidase performs hydrolytic cleavage between MurNAc and GlcNAc residues, whereas lytic transglycosylases (LTs) carry out non-hydrolytic cleavage, forming an intermolecular ether bond yielding a non-reducing 1,6-anhydroMurNAc (anhMurNAc) residue (9, 10). (iii) Endopeptidases cleave non-terminal amino acids from stem peptides or cross-links between dimuropeptides (2), and terminal amino acids are cleaved by carboxypeptidases (11).

*Escherichia coli* recycles 40%–50% of its cell wall constituents per generation (12, 13). The PG fragments released by the action of PG degradative enzymes are either released into the environment or transported back into the cytoplasm for PG recycling (14). The PG recycling pathway has been well documented in some gram-negative bacterial model systems (15). In several gram-negative bacteria, including *E. coli* and *Enterobacter* spp., anhydromuropeptides generated by the action of LTs are transported across the inner membrane by the permease AmpG (16). Inside the cytoplasm, the anhydromuropeptides are processed by various enzymes. Terminal D-Ala is cleaved by LD-carboxypeptidase LdcA (17). AmpD (cytosolic *N*-acetyl-anhydromuramyl-l-alanine amidase) removes the stem peptide (18), and NagZ acts on sugar residues, separating GlcNAc from anhMurNAc (16, 19). AnmK processes anhMurNAc to generate MurNAc-6-phosphate, ultimately forming UDP-MurNAc (20). Mpl ligates the peptides onto UDP-MurNAc, which are then utilized for PG synthesis (21). Recent studies have shown that PG recycling takes place in different gram-positive bacteria as well (22, 23). This process may support bacterial viability under starvation conditions (22–24) and contributes to resistance against antibiotics and lysozyme (25–27). However, the detailed mechanisms by which these bacteria reuse PG sugars and peptide turnover products for PG synthesis remain poorly understood.

In some gram-negative bacteria, anhMurNAc-peptides also act as a signaling molecule by regulating the induction of the β-lactamase gene (14). In *Enterobacter* spp., *Pseudomonas aeruginosa*, and *Agrobacterium tumefaciens*, anhMurNAc also acts as transcriptional activators of the inducible β-lactamase gene *ampC* (28, 29). In these bacteria, AmpC expression is modulated by a LysR-type transcription regulator AmpR (30). AmpR controls both activation and repression of AmpC, and anhMurNAc muropeptides released by the action of LTs activate AmpR (31, 32). In contrast, binding cell wall precursor molecules such as UDP-MurNAc pentapeptides (UDP-MurNAc-P5) to AmpR represses the transcription of *ampC* (31). Inhibition of cell wall synthesis by β-lactams leads to the accumulation of anhydromuropeptides in the cytoplasm, activation of AmpR, and increased *ampC* expression (31). However, *ampC* expression is not regulated by AmpR in some bacteria like *E. coli, Acinetobacter baumannii,* and *Shigella* spp. (33–35).

LTs are integral to peptidoglycan recycling and turnover in gram-negative bacteria (36). Some examples representing the relation between LTs and antibiotic resistance include *Shewanella oneidensis*, a bacterium known for its ability to reduce metal ions, which has increased β-lactam resistance in its LTs SltY, MltB, and MltB2 deletion mutants (37). In *Campylobacter jejuni*, a mutation in its putative LTs *cj0843c* leads to elevated β-lactam resistance (38). Lack of *slt70*, *mltA*, and *mltB* in *E. coli* hinders PG turnover, further inhibiting the induction of AmpC β-lactamase (39). MltB3 of *A. tumefaciens* C58 also contributes toward AmpC-dependent β-lactamase induction (29). Besides their central role in regulating cell morphology, division, and β-lactamase induction, LTs of *Vibrio cholerae* reduce adverse effects of periplasmic accumulation of PG polymers, and hence, the presence of LT is essential for cell survival (40). *Caulobacter crescentus* genome encodes four characterized LTs—SdpA, SdpB, SdpC, and PleA (41). Localization of *sdpA* and *sdpB* to midcell depends on FtsN (41). Recent research has shown that the deletion of a single *sdpA* gene enhances sensitivity toward ampicillin along with cell shape defects in *C. crescentus* (41).

In this study, we investigated the role of PG recycling genes on β-lactam resistance in *C. crescentus*. We found homologs of several PG recycling genes in *C. crescentus*. Analysis of PG turnover products revealed that ∆*ampG* deletion mutant accumulates anhydromuropeptides in the periplasm. Additionally, both ∆*sdpA* and ∆*ampG* mutants are sensitive toward β-lactams and exhibit morphological aberration and cell lysis. While the absence of SdpA or AmpG does not lead to adverse effects, the combined inactivation of these two genes strongly reduces cell viability. Moreover, the enhanced activity of either SdpA or AmpG also caused morphological aberrations. Precursor analysis of PG recycling mutants revealed decreased soluble PG precursors UDP-MurNAc, UDP-GlcNAc, and UDP-MurNAc-pentapeptide (UDP-MurNAc-P5). Furthermore, growth media supplemented with GlcNAc were able to mitigate antibiotic sensitivity in ∆*sdpA* and ∆*ampG* mutants, suggesting that cell wall turnover products may be employed in *de novo* PG biosynthesis. Collectively, our study reveals that PG turnover products are recycled in *C. crescentus,* and perturbations in PG recycling cause deficiencies in cell wall biogenesis, leading to antibiotic sensitivity.

## RESULTS

### The absence of *sdpA* leads to β-lactam sensitivity

**Fig 1:**
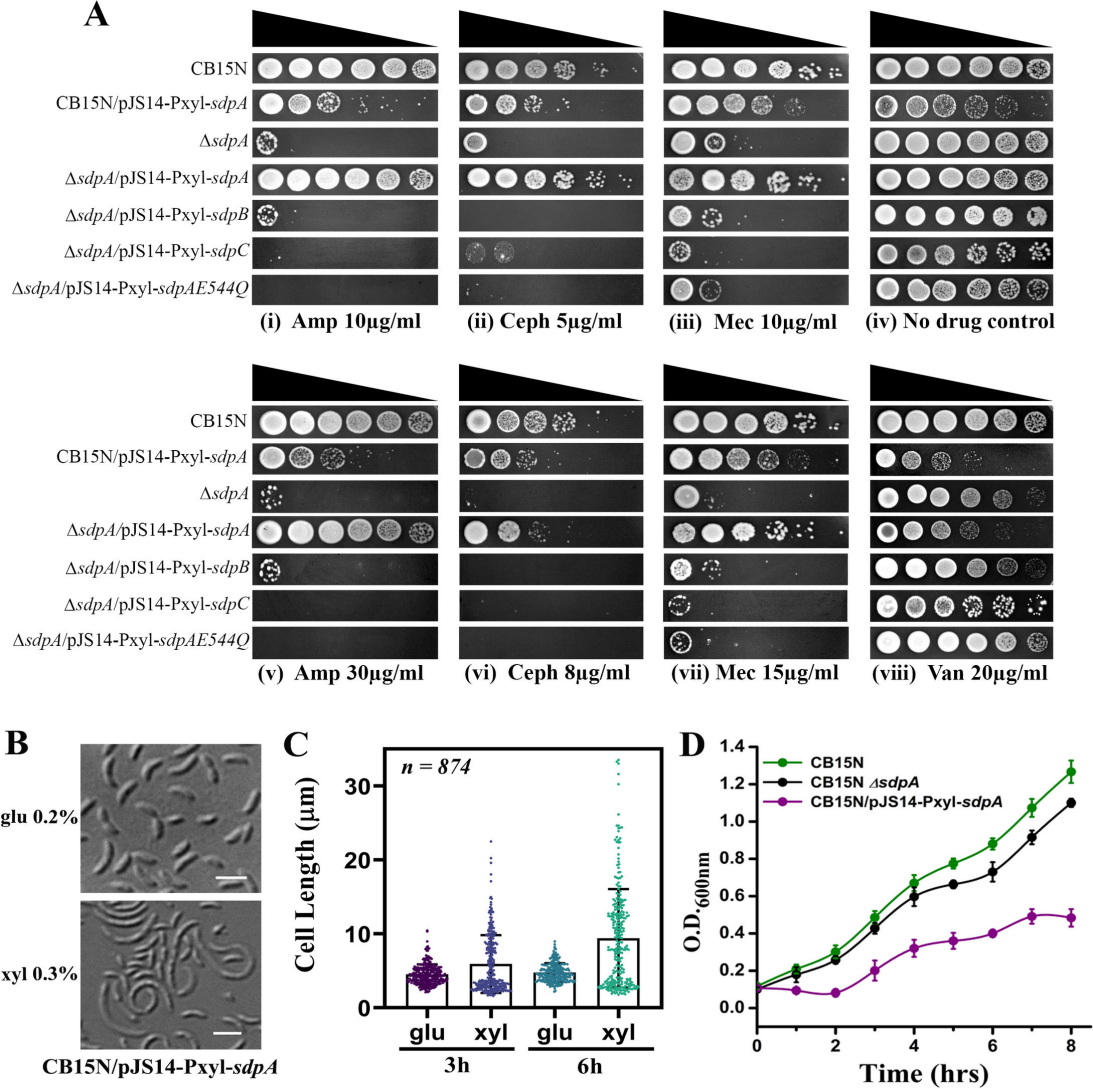
(**A**) Role of SdpA in β-lactam resistance. β-Lactam antibiotic susceptibility assay was performed via spotting dilutions. Diluted cultures (6 µL) of strains CB15N, RP48 (CB15N/pJS14-Pxyl-*sdpA*), RP47 (∆*sdpA*), RP52 (∆*sdpA*/pJS14/Pxyl/*sdpA*), RP53 (∆*sdpA*/pJS14/Pxyl/*sdpB*), RP54 (∆*sdpA*/pJS14/Pxyl/*sdpC*), and RP55 (∆*sdpA*/pJS14/Pxyl/*sdpAQ544E*) were spotted on PYE + 0.3% xylose containing antibiotics at varying concentrations (ampicillin 10 and 30 µg/mL, cephalexin 5 and 8 µg/mL, mecillinam 10 and 15 µg/mL, vancomycin 20 µg/mL). PYE plates with no antibiotic were used as a control. All plates were incubated at 30°C for 36 h before imaging. (**B**) Overexpression of SdpA leads to morphological and cell division defects. Strain RP48 (CB15N/pJS14-Pxyl-*sdpA*) was grown to the exponential phase with 0.3% xylose added to the media. The culture was immobilized on a PYE agarose pad and imaged by DIC microscopy after 6 h post-induction at 30°C (scale bar = 2 µm). (**C**) Distribution of cell length in a population of RP48 after the overexpression of *sdpA*. Micrographs of strain RP48 were subjected to automated image analysis by Oufti (45). The data obtained (*n* = 874 cells per condition) are shown as a scatter plot where the bar represents the median. (**D**) Growth rate of the *sdpA* overexpression (induced with xylose) strain compared to CB15N and *sdpA* deletion strain was analyzed. OD_600_ was measured at 2 h interval for a total period of 8 h. Data represent the mean of three biological replicates.

*C. crescentus* displays high levels of tolerance toward β-lactams (1). *C. crescentus* encodes three soluble lytic transglycosylases (SLT) domain proteins, SdpA/B/C (41), which are predicted to be soluble periplasmic proteins. A previous study showed that SdpA was essential for β-lactam resistance (41), and deletion of *sdpA* rendered mutants susceptible to ampicillin, whereas the absence of *sdpB* or *sdpC* did not affect ampicillin sensitivity (Fig. 1) (41). Similarly, our results demonstrate that *sdpA* mutants (RP47) have decreased viability in ampicillin (Fig. 1A i, v). Further investigation revealed that *sdpA* mutants were also fitness compromised under cephalexin (Fig. 1A–ii, vi) and mecillinam (Fig. 1A–iii, vii) treatment. Ectopic expression of *sdpA* from pJS14 plasmid restored β-lactam resistance to wild-type (WT) levels (Fig. 1A). In *E. coli* Slt70, Glu-478, an active site residue at the C-terminal, is essential for its catalytic activity (42, 43). To probe whether the LT activity of SdpA is required for β-lactam resistance, an active site mutant of *sdpA*, lacking the essential glutamic acid at the active site 544, was generated (Fig. S1). The active site variant could not complement and restore β-lactam resistance in the *sdpA* deletion background (Fig. 1A), suggesting that the LT activity of SdpA is essential for antibiotic tolerance. Furthermore, ectopic expression of *sdpB* and *sdpC* from pJS14 plasmid could not restore antibiotic resistance in *sdpA* mutants (Fig. 1A), indicating a central and specific role of SdpA. While CB15N shows high resistance to vancomycin, to our surprise, *sdpA* mutants were comparatively less fitness compromised in the presence of vancomycin (20 µg/mL) (Fig. 1A–viii), probably indicating that the integrity of the outer membrane is not perturbed in ∆*sdpA*. Similarly, single LT mutants do not display increased sensitivity to vancomycin in *P. aeruginosa* (44). Collectively, our results indicate that *sdpA* mutants are sensitive to β-lactam antibiotics.

### Overexpression of SdpA causes morphological abnormalities

While performing a spot assay test, we observed that the overexpression of SpdA in wild-type background led to a decrease in cell viability (Fig. 1A). To probe this phenotype further, RP48 (CB15N/pJS14-P*xyl-sdpA*) cells expressing *sdpA* under the control of xylose-inducible promoter in wild-type background were cultured in media supplemented with 0.3% xylose and were imaged by DIC microscopy. As depicted in Fig. 1B, after 4 h, cell division was arrested, resulting in a filamentous growth. Additionally, after 8 h of growth in the presence of xylose, cell death was observed (Fig. 1B). Image analysis revealed a significant increase in cell length (*l* = 9.38 µm ± 0.94 µm, mean ± standard deviation) in xylose-supplemented media compared to control cells grown under glucose conditions (*l* = 3.81 µm ± 0.77 µm) (Fig. 1C). Along with morphological defects, the prolonged overexpression of *sdpA* decreased cell viability (Fig. 1D).

As cells overexpressing SdpA exhibited impaired cell division, we further investigated which divisome components were affected in these cells. SdpA is known to interact with DipM and FtsN (36, 40, 41, 46). FtsN localization was investigated in the SdpA overexpression background. In RP61 cells, induction with xylose resulted in cell elongation and failure of FtsN to localize to new divisome sites (Fig. S2A and B). In comparison, cells grown in the presence of glucose displayed medial FtsN localization (Fig. S2A and B). To further investigate the effect of *sdpA* overexpression on the localization pattern of other divisome proteins, we examined the localization pattern of FtsZ. Distinct YFP-FtsZ foci were observed in elongated cells (RP62), suggesting that FtsZ localization is not perturbed due to *sdpA* overexpression. (Fig. S2C). Together, our data suggest that morphological defects due to *sdpA* overexpression are probably due to the mislocalization of FtsN.

**Fig 2:**
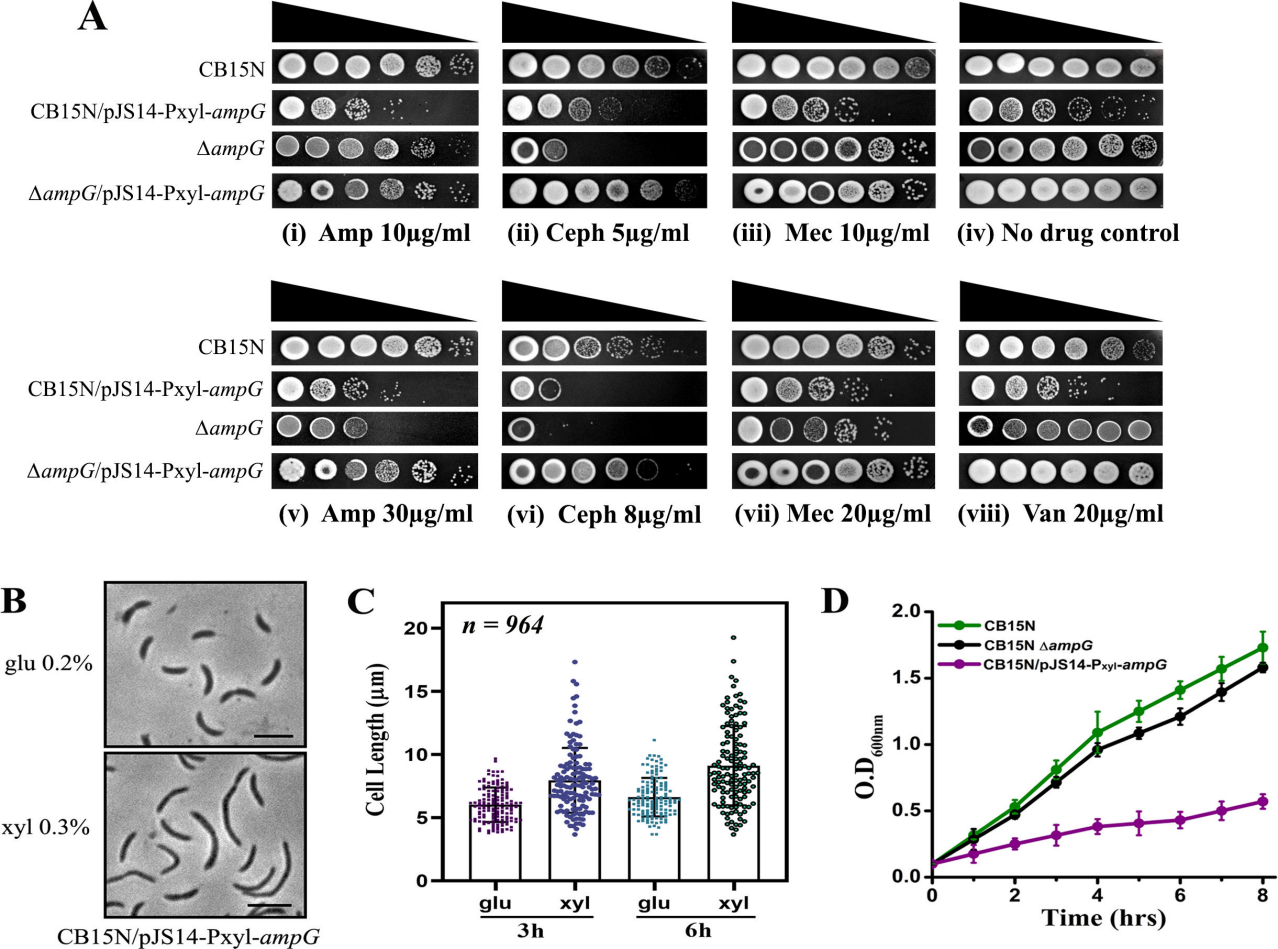
(**A**) Role of AmpG permease in β-lactam resistance. β-Lactam susceptibility assay was performed by spotting dilutions of cultures CB15N, RP57 (CB15N/pJS14-Pxyl-*ampG*), RP56 (∆*ampG*), and deletion mutant along with its complemented derivative RP58 (∆*ampG*/pJS14-Pxyl-*ampG*) on PYE solid media + 0.3% xylose containing antibiotics at varying concentrations. PYE plates with no antibiotic were used as a control. (**B**) Phase contrast imaging of *ampG* overexpression strain RP57 (CB15N/pJS14-Pxyl-*ampG*) showing cell filamentation. Cells were grown to mid-exponential phase for 6 h in a medium containing (+) or lacking (–) xylose and were imaged by phase contrast microscopy (scale bar = 2 µm). (**C**) Distribution of cell length in a population of RP57 after the overexpression of AmpG. Micrographs from (**B**) were then subjected to automated image analysis by Oufti software. The values obtained are shown as scatter plots, where the bar indicates the median (*n* = 964 per condition). (**D**) Growth rate of strains CB15N, RP56 (∆*ampG*), and RP57 (CB15N/pJS14-Pxyl-*ampG*) confirmed growth defects. Cells were induced with xylose at mid-exponential phase, and OD_600_ was measured at a 2 h interval at 30°C. Data represent the mean of three biological replicates.

### AmpG mutants are sensitive to β-lactams

The muropeptide recycling pathway in *C. crescentus* has yet to be delineated. In many bacteria, anhydromuropeptides generated by the action of lytic transglycosylase are selectively transported to the cytoplasm via a permease, AmpG (16). Recent studies have shown AmpG to be involved in antibiotic resistance in many bacteria, such as *E. coli* (47), *Klebsiella aerogenes* (48), *P. aeruginosa* (49), and *Citrobacter freundii* (50), while others, such as *A. tumefaciens,* use YejBEF-YepA (51) instead. Our initial screening revealed that the *C. crescentus* genome encodes an ortholog of *ampG (CC_0137*). To test whether *ampG* plays a role in antibiotic tolerance, an *ampG* deletion mutant (RP56) was created. CB15N cells lacking *ampG* displayed no aberrant morphological or growth defects (Fig. S3), suggesting it to be dispensable for growth under standard laboratory conditions. However, Δ*ampG* mutants were sensitive to β-lactam antibiotics (Fig. 2A). While ∆*ampG* cells were sensitive toward β-lactam antibiotics, they showed higher tolerance to antibiotic concentrations compared to Δ*sdpA*. Δ*sdpA* mutants displayed growth defects at 10 µg/mL of ampicillin, while ∆*ampG* mutants could grow at 10 µg/mL of ampicillin (Fig. 2A–i) but were sensitive at 30 µg/mL (Fig. 2A–v) or higher. Similarly, in cephalexin, Δ*ampG* mutants displayed growth defects at a concentration of 8 µg/mL or higher (Fig. 2A–ii, vi), and could tolerate mecillinam up to 20 µg/mL (Fig. 2A–iii, vii). The antibiotic sensitivity was due to the absence of *ampG*, as the ectopic expression of *ampG* from pJS14 plasmid restored antibiotic resistance to wild-type levels (Fig. 2A). Similar to ∆*sdpA,* ∆*ampG* mutants were resistant to vancomycin (Fig. 2A–viii). Further microscopic examination of CB15N cells overexpressing AmpG revealed morphological defects. Cells RP57 (CB15N/pJS14-P*xyl-ampG*) grown in the presence of xylose (inducer) were filamentous, exhibiting cell division defects (Fig. 2B); in contrast, cells grown in glucose repressed *ampG* expression and had normal cell morphology (Fig. 2B). Continuous growth in the presence of xylose led to an increase in cell length (*l* = 9.04 µm ± 0.37 µm) and appearance of filamentous morphology, indicating impaired cell division (Fig. 2C). Growth curve analysis further corroborated that AmpG-overexpressing cells have severe growth and division defects (Fig. 2D).

### AmpG displays punctate pattern localization in the cell membrane

**Fig 3:**
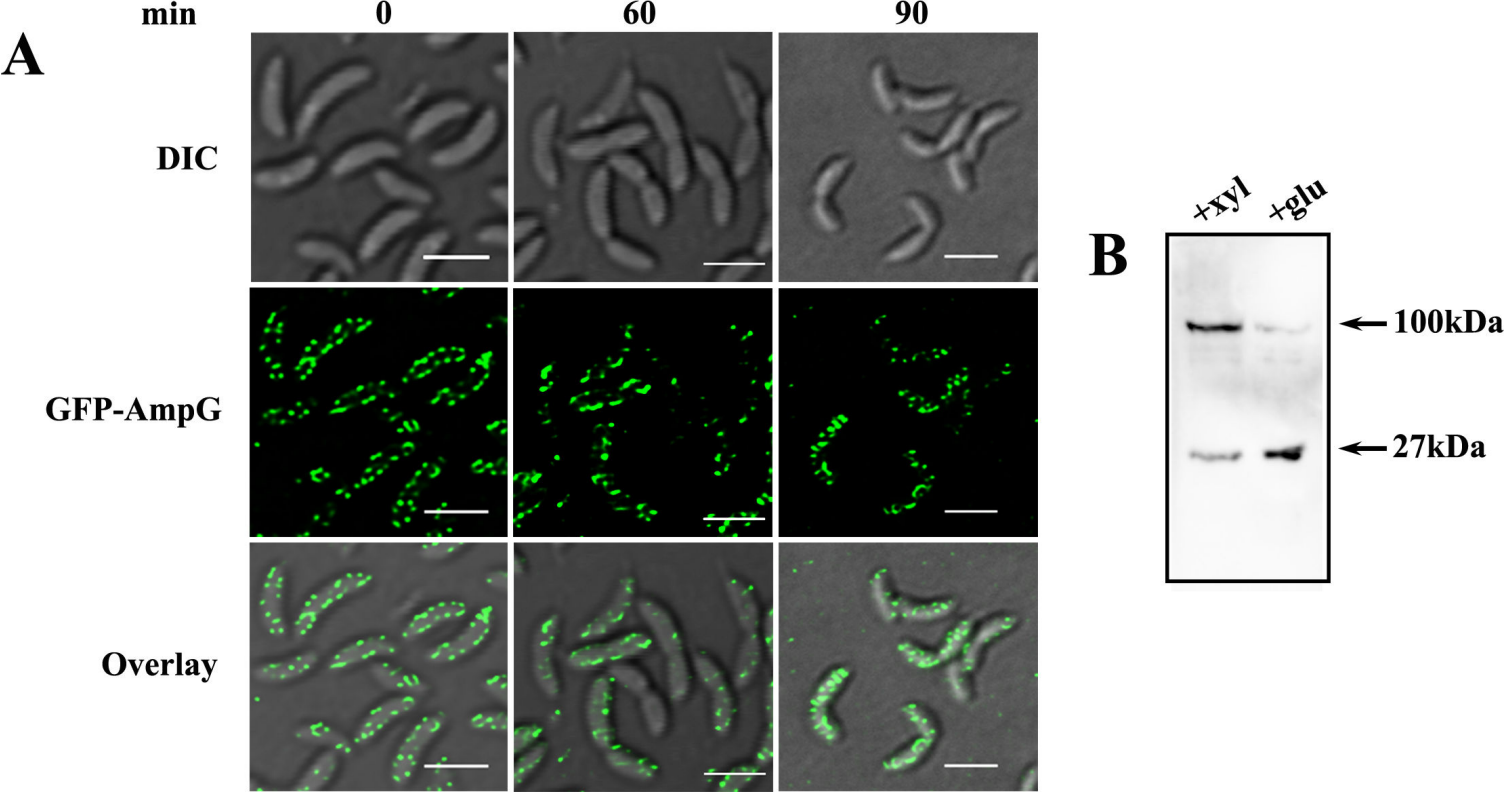
(**A**) Localization of AmpG permease during *Caulobacter* cell cycle. DIC and fluorescence microscopy images of cells RP72 with *ampG* tagged with GFP fusion protein under the control of xylose promoter. The cells were synchronized, and samples were removed at different time points and imaged in a confocal microscope (scale bar = 2 µm). (**B**) Western blot to confirm the stability of GFP-*ampG* fusion protein. Full-length GFP-*ampG* fusion protein (~100 kDa) expressed under the control of xylose promoter probed with anti-GFP antibody. The 27 kDa band represents the free GFP.

To investigate the localization of AmpG in *C. crescentus* cells, N-terminus GFP-tagged AmpG fusion (RP72) was generated. CB15N cells expressing GFP-AmpG fusion protein were synchronized and observed by confocal microscopy. GFP-fused *ampG* was localized in a patchy pattern in the cell membrane along the entire length of the cell (Fig. 3A). The GFP-AmpG did not accumulate at the division site as no mid-cell localization was observed even in dividing cells (Fig. 3A). To confirm the localization pattern of GFP-AmpG is not due to the cleavage of chimeric protein, Western blot analysis was performed using α-GFP antibodies. Western blot analysis validated the presence of a stable full-length GFP-AmpG fusion protein in cells grown in the presence of xylose, and a negligible level of free GFP was detected compared to glucose control (Fig. 3B). The exclusion of GFP-AmpG from division sites probably indicated that AmpG may not be essential for cell division in *C. crescentus*.

### SdpA and AmpG mutants display defects in PG synthesis under β-lactam stress

**Fig 4:**
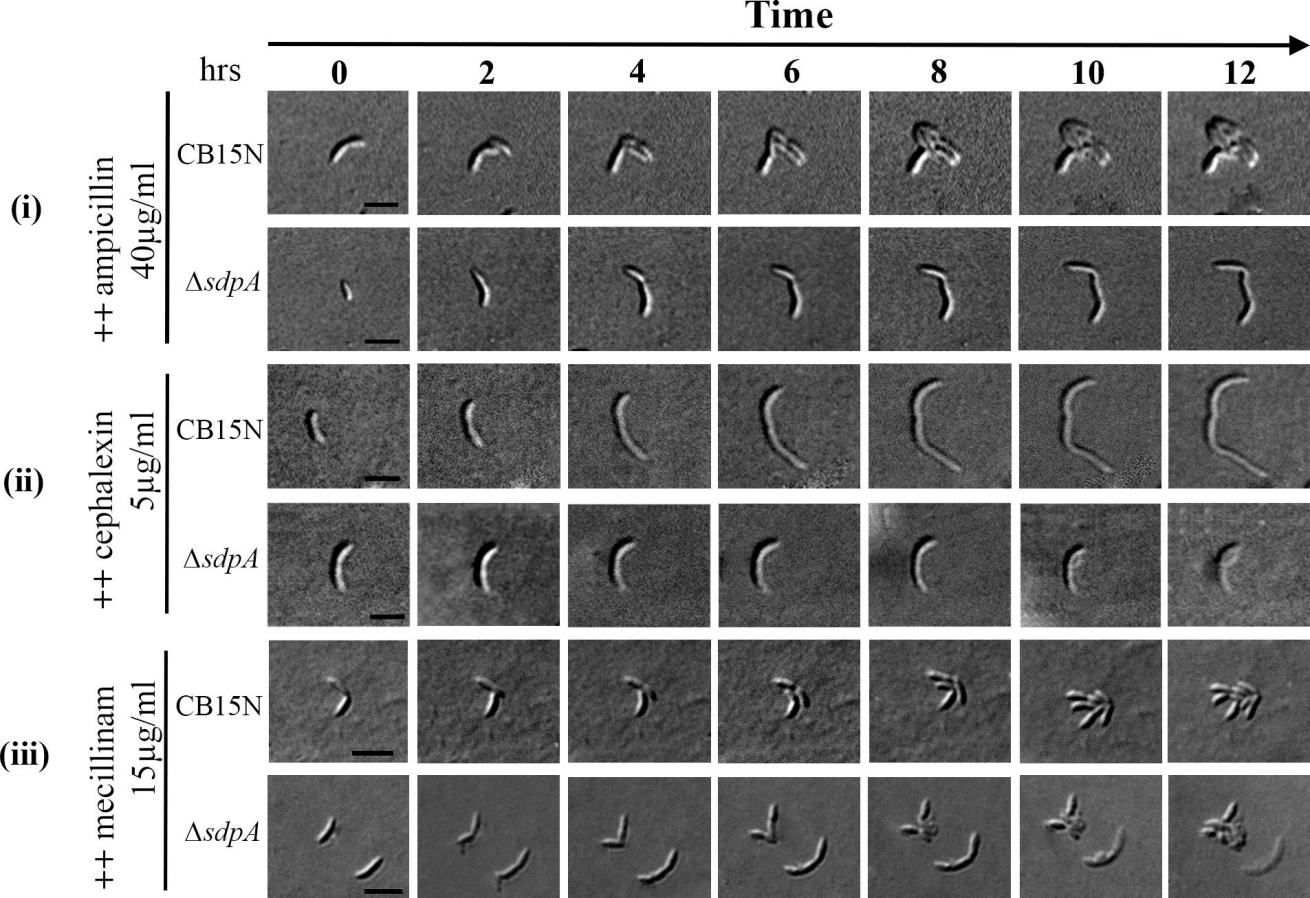
Time-lapse imaging of strains CB15N (WT) and RP47 ∆*sdpA* in the presence of β-lactam drugs—ampicillin (40 µg/mL), cephalexin (5 µg/mL), and mecillinam (15 µg/mL). Overnight cell cultures were diluted in PYE media and allowed to grow for 2 h at 30°C, and then cultures were immobilized on PYE agarose pad supplemented with β-lactam drugs and were imaged for up to 12 h at 30°C (scale bar = 2 µm). Corresponding movies for each panel are in aupplemental materials (Movies S1–S6)

Time-lapse microscopy was performed to investigate how *sdpA* mutants behave under antibiotic stress. Wild-type cells grew and divided for 10 h when treated with 30 µg/mL ampicillin. In contrast, cells lacking *sdpA* were compromised for cell division and formed filaments (Fig. 4). Treatment with cephalexin, an inhibitor of PBP3 (52, 53), caused *C. crescentus* cells to elongate into long filaments (Fig. 4). Conversely, Δ*sdpA* mutants treated with cephalexin initially grew slowly for 3 h, followed by growth cessation and eventual lysis (Fig. 4). Similarly, Δ*sdpA* cells ceased growth and lysed in the presence of mecillinam (Fig. 4).

**Fig 5:**
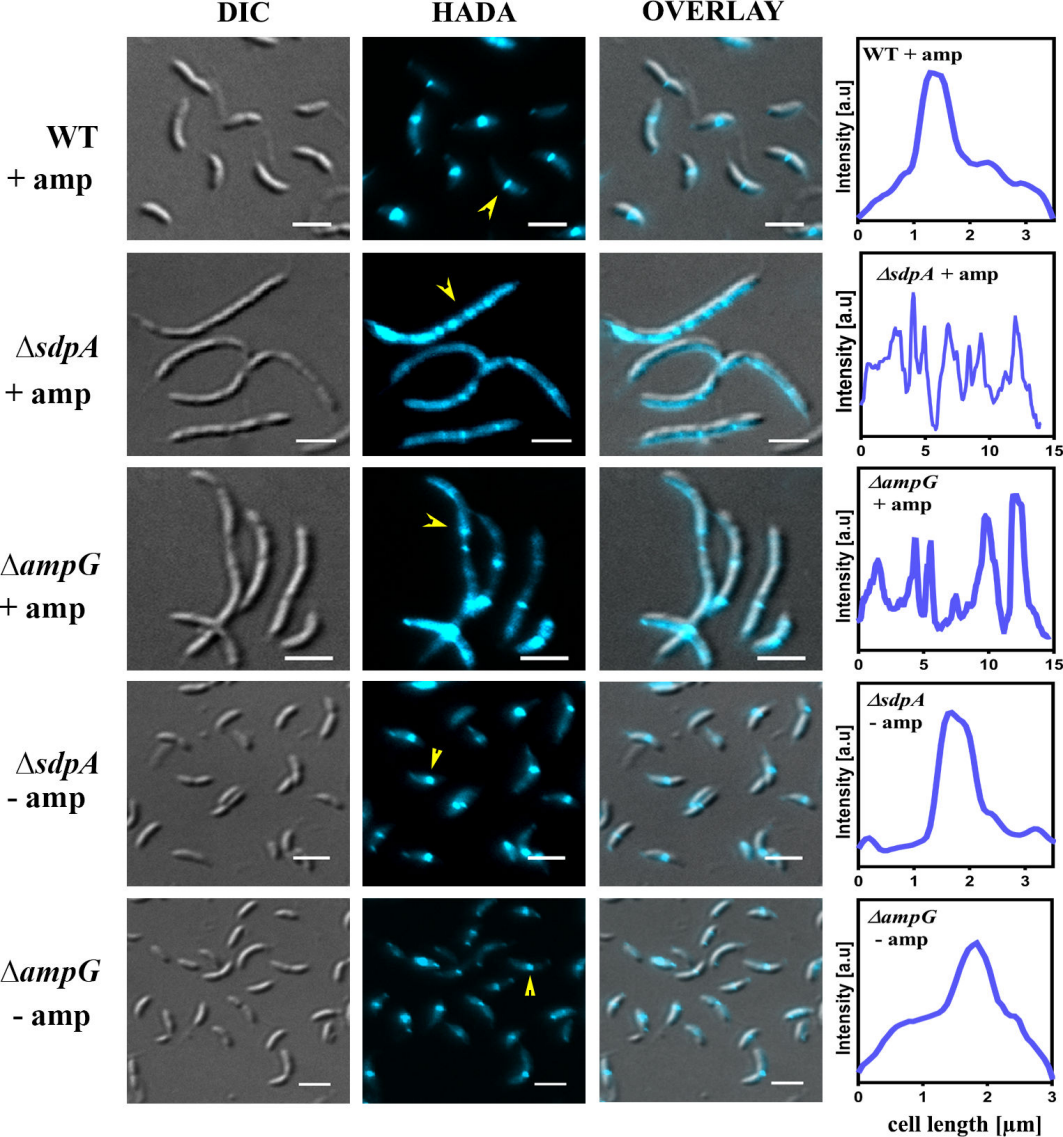
Ampicillin affects the incorporation of newly synthesized cell wall in PG recycling mutants. HADA labeling of strains CB15N, RP47 (∆*sdpA*), and RP56 (∆*ampG*) in the presence (+ amp) and absence (– amp) of ampicillin. Overnight cultures were diluted in PYE media, grown to 0.1 OD_600_, and treated with 40 µg/mL ampicillin for 4 h. Cells were collected, washed with minimal media, and incubated with 0.5 mM HADA for 15 min at 30°C. Cells were washed with minimal media again and imaged using a Nikon Fluorescence Microscope CFP filter. A representative cell (yellow arrowheads) was selected from each strain condition shown, and their fluorescence profiles were extracted along the cell length axis. Fluorescence intensity (arbitrary units [a.u.]) is plotted against the position along the entire cell length (scale bar = 3 µm).

We further investigated defects in PG synthesis utilizing fluorescent D-amino acid hydroxycoumarin-carbonyl-amino-D-alanine (HADA), which labels active PG synthesis sites. HADA labeling was performed in WT*,* ∆*sdpA*, and ∆*ampG* cells under ampicillin treatment. Wild-type cells exhibited distinct bands of HADA labeling at midcell, indicating new PG incorporation (Fig. 5). In contrast, ∆*sdpA* and ∆*ampG* cells, which were filamentous under ampicillin treatment, showed multiple diffused bands (Fig. 5), suggesting perturbation in PG synthesis. As a control, we also examined ∆*sdpA* and ∆*ampG* cells without antibiotic exposure, and in both cases, HADA labeling was observed at midcell (Fig. 5). Line scan analysis representing fluorescence intensity profile along the cell length revealed multiple high-intensity peaks of HADA signal in ∆*sdpA* and ∆*ampG* mutants under ampicillin treatment. In contrast, untreated cells displayed the highest peak intensity only closer to midcell (Fig. 5). Our results indicate that ∆*sdpA* and ∆*ampG* mutants display cell wall synthesis defects under β-lactam stress.

### Anhydromuropeptides accumulate in the periplasmic space in the absence of AmpG

**Fig 6:**
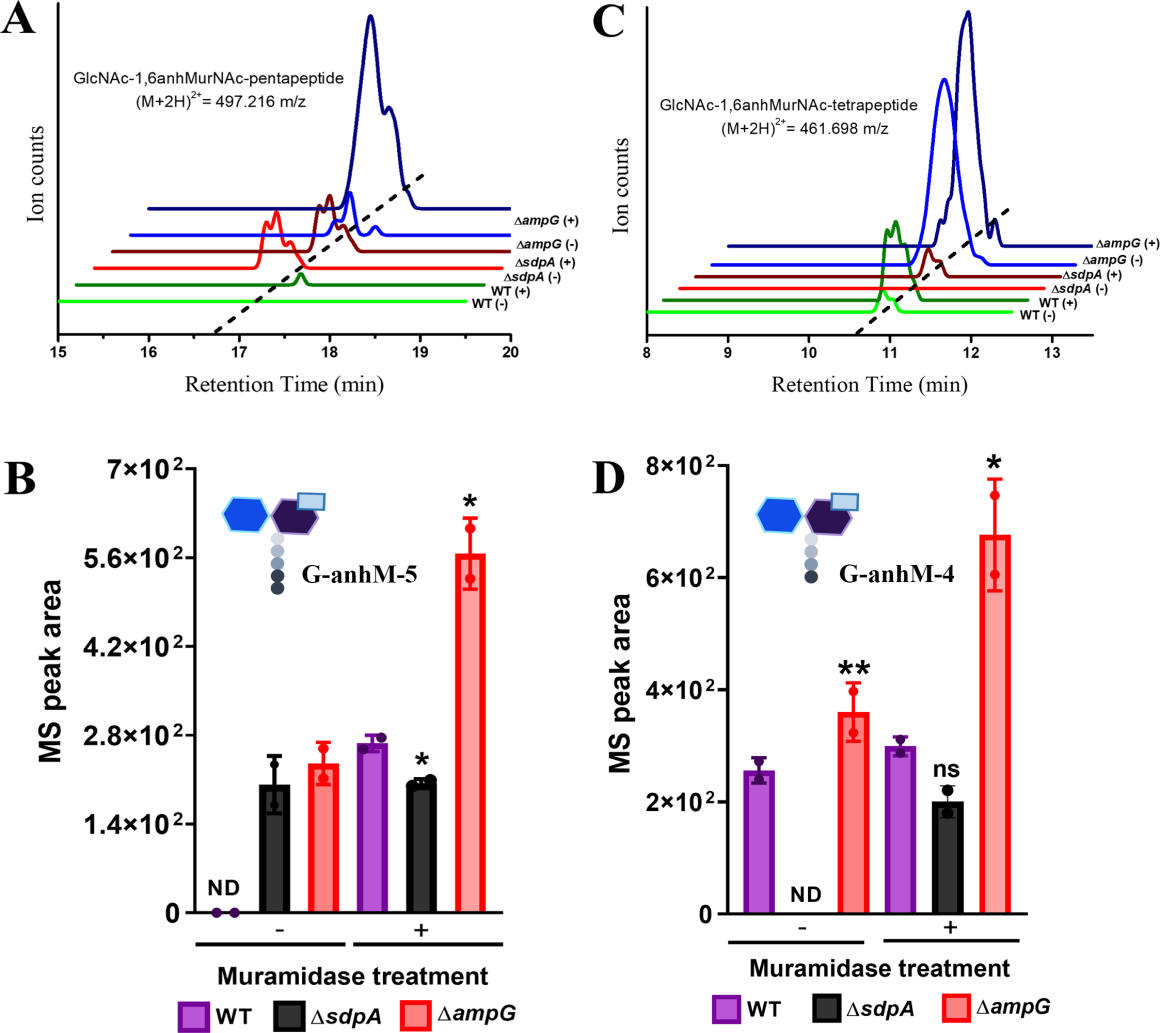
AmpG deletion leads to periplasmic crowding of anhydromuropeptides. Cultures of strains CB15N, RP47 (∆*sdpA*), and RP56 (∆*ampG*) were grown up to OD_600_ 0.6 and harvested for LC-MS analysis of periplasmic fraction of PG recycling mutants. (**A, B**) Representative LC-MS chromatograms for GlcNAc-anhMurNAc-P5 (M+2H)^2+^ = 497.216 *m*/*z* and GlcNAc-anhMurNAc-P4 (M+2H)^2+^ = 461.698 *m*/*z* for mutants CB15N, RP47 (∆*sdpA*), and RP56 (∆*ampG*). (**C, D**) Quantification of anhydromuropeptides level (GlcNAc-anhMurNAc-P5 and GlcNAc-anhMurNAc-P4) in PG recycling mutants using the integrated peak intensity. Error bars represent the standard deviation from two biological replicates. ND, not determined (*P < 0.05, **P < 0.005, ***P < 0.005, ****P < 0.0005 by *t*-test between WT vs RP47 and RP56).

Anhydromuropeptides generated by the action of SdpA are transported into the cytoplasm by AmpG, and *ampG* deletion should lead to accumulation of anhydromuropeptides in the periplasmic space. To investigate this further, we isolated periplasmic soluble muropeptides from sedimented purified sacculi, followed by muramidase digestion and analysis by liquid chromatography-mass spectrometry (LC-MS). Both muramidase-treated and untreated samples were compared to identify monomeric muropeptide accumulation in the periplasm of PG recycling mutants. In wild-type cells, both GlcNAc-1,6-anhMurNAc-pentapeptide (G-anhM-5) (Fig. 6A and B; Table S2) and GlcNAc-1,6-anhMurNAc-tetrapeptide (G-anhM-4) (Fig. 6C and D; Table S2) were detected at low levels upon muramidase treatment (Fig. 6). There was no significant difference observed in the levels of G-anhM-5 and G-anhM-4 between ∆*sdpA* mutant and wild type (Fig. 6). This could be because of the generation of anhydromuropeptides by other lytic transglycosylase enzymes in the absence of *sdpA*. In contrast, a significant accumulation of G-anhM-5 and G-anhM-4 was observed in *ampG* deletion background (Fig. 6), indicating a failure in transporting anhydromuropeptides to the cytoplasm. Taken together, these results indicate that AmpG facilitates PG recycling by transporting anhydromuropeptides into the cytoplasm in *C. crescentus*.

### β-Lactamase activity is not perturbed in *sdpA* and *ampG* mutants

**Fig 7:**
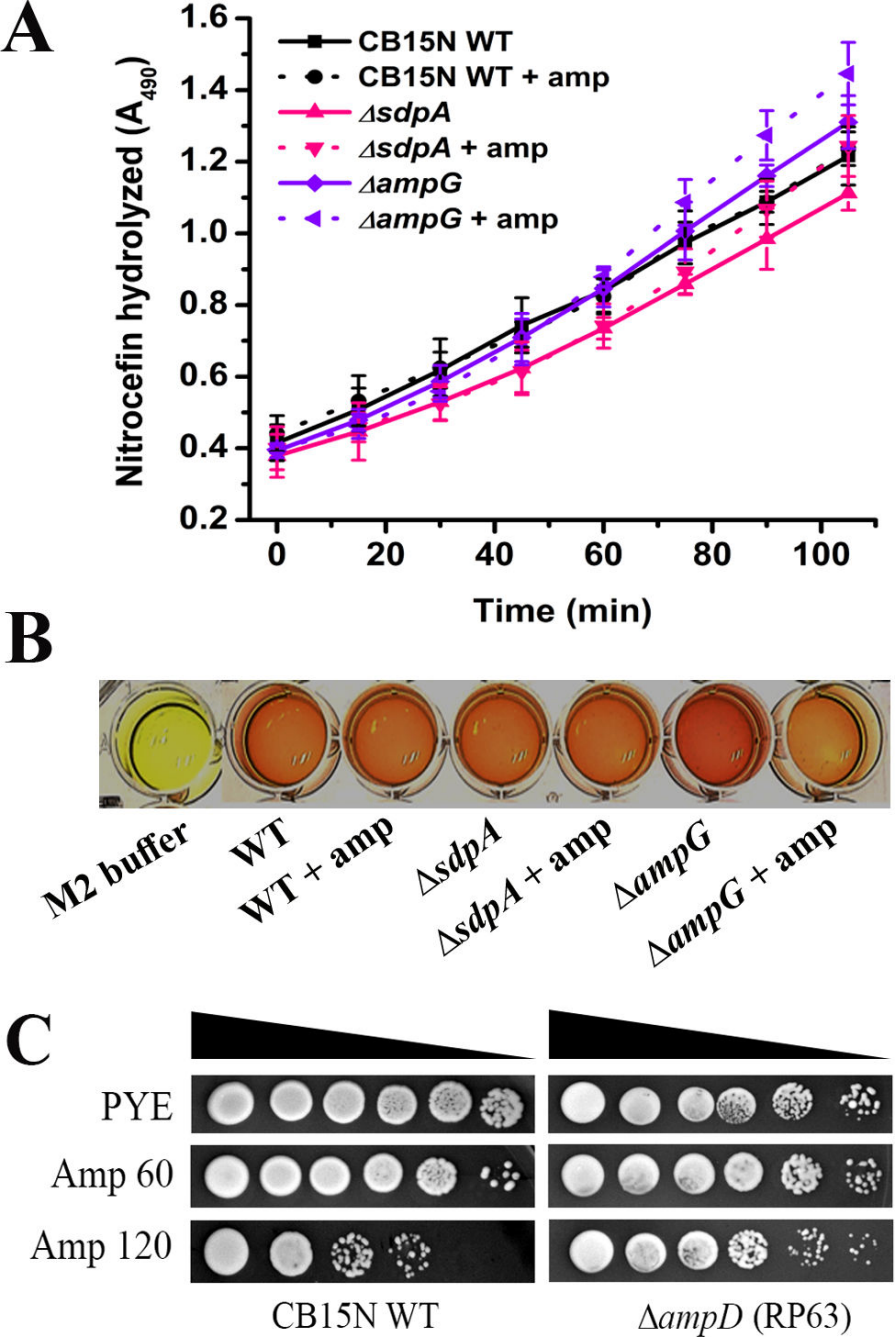
(**A**) Spectrophotometric assay representing nitrocefin degradation. Cultures of strains NA1000 (WT), RP47 (∆*sdpA*), and RP56 (∆*ampG*) were grown to OD_600_ and then cultivated for 3 h in the presence (+ amp) or absence (– amp) of 10 µg/mL ampicillin. Hydrolyzed nitrocefin substrate was measured by monitoring absorbance at 486 nm at regular intervals. The data shown represent one of three biological replicates. (**B**) Cell lysates of WT, RP47 (∆*sdpA*), and RP56 (∆*ampG*) with and without ampicillin treatment were incubated with nitrocefin solution for 30 min. M2 buffer was used as a negative control. The appearance of red color product indicates a positive β-lactamase activity, while a consistent yellow color indicates the absence of activity. (**C**) Inactivation of AmpD resulted in elevated β-lactam resistance. Ampicillin susceptibility assay by spot dilutions. The optical density of overnight cultures of strains CB15N and RP63 (∆*ampD*) were normalized to 1.0, and cultures were spotted on PYE + 0.3% xylose plates with and without ampicillin at varying concentrations (60 and 120 µg/mL) and were incubated for 48 h at 30°C.

*C. crescentus* is intrinsically resistant to β-lactams due to the activity of potent β-lactamase (Mbl1B) (54). Interestingly, deletions of LTs did not affect the expression and activity of Mbl1B, suggesting that PG recycling fragments may not regulate the expression of intrinsic β-lactamase in *C. crescentus* (41). A nitrocefin hydrolysis assay was performed to test whether the β-lactam sensitivity of *sdpA* and *ampG* mutants was not due to decreased β-lactamase activity. Both *sdpA* and *ampG* mutants showed similar β-lactamase activity as wild-type cells upon ampicillin treatment, suggesting that the antibiotic sensitivity of these mutants is not due to decreased β-lactamase activity (Fig. 7A and B).

**Fig 8:**
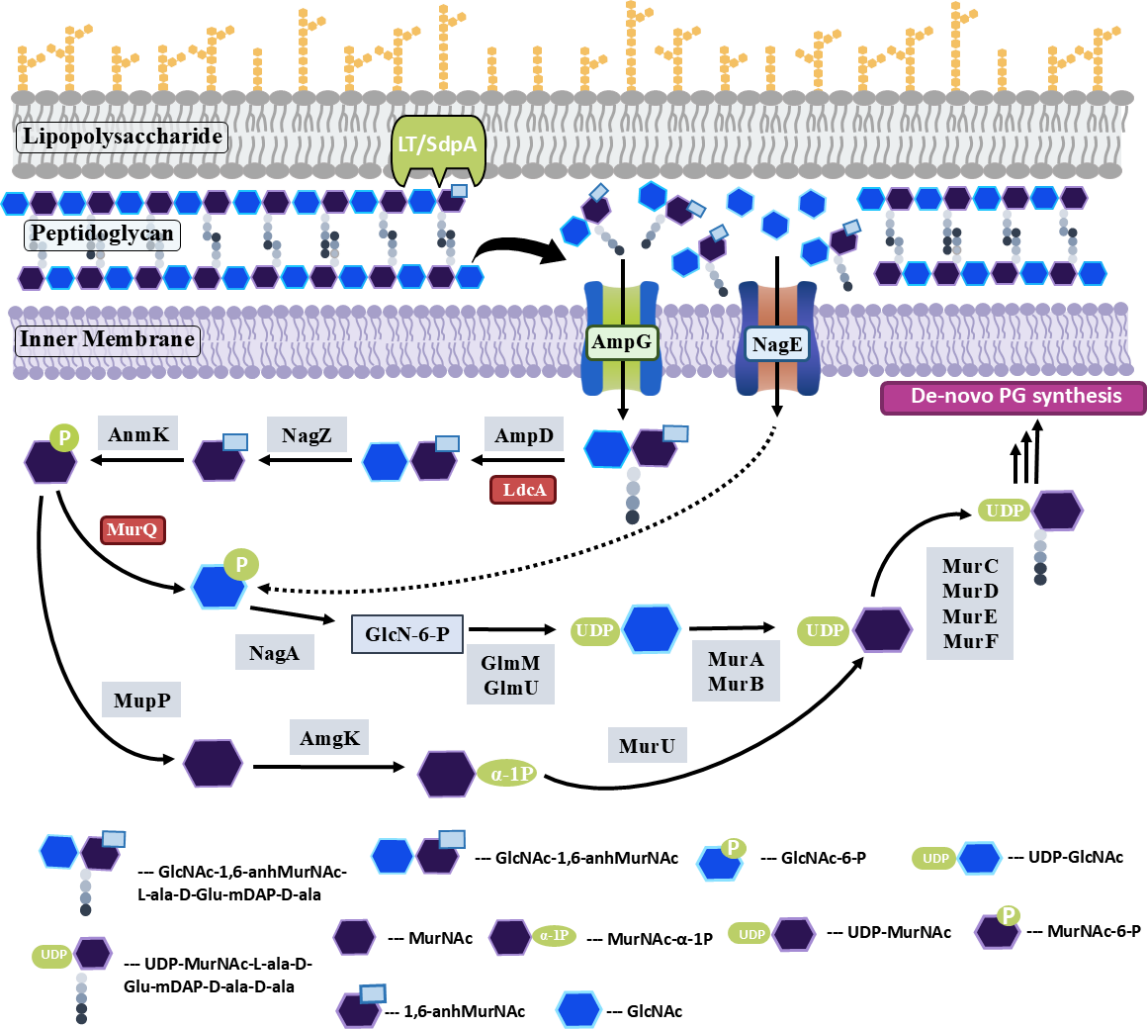
Schematic representation of the proposed PG recycling pathway in *C. crescentus*. Anhydromuropeptides released by the enzymatic activity of LTs are transported into the cytoplasm by membrane transporters AmpG and NagE, which are then processed by various downstream proteins. (Recycling genes not found in *C. crescentus* are marked in red box.)

In PG recycling, cell wall degradation products are transported into the cytoplasm by AmpG and further processed by the AmpD and NagZ enzymes. We found orthologs of both AmpD and NagZ in *C. crescentus* (Table 1; Fig. 8). AmpD is a cytoplasmic amidase that removes the stem peptide sugar moiety (18), comprising the *N*-acetylmuramoyl-L-alanine amidase domain of Type 2 (NLAA-2) (55). AmpD specifically cleaves the stem peptide from 1,6-anhydromuromyl residues (56) as shown in several gram-negative bacteria. In *Enterobacter cloacae*, *C. freundii*, and *P. aeruginosa,* it has been shown that the inactivation of AmpD leads to enhanced β-lactamase (AmpC) expression (57, 58). *C. crescentus* AmpD contains an amidase catalytic domain and a PG-binding domain. To our surprise, the *ampD* deletion mutant (RP63) was not sensitive to ampicillin (Fig. 7C). Δ*ampD* cells could tolerate 120 µg/mL ampicillin (Fig. 7C). In contrast, wild-type cells were fitness compromised at this concentration (Fig. 7C), suggesting that *ampD* may not be crucial for β-lactam resistance in *C. crescentus*.

**TABLE 1:** List of PG recycling genes in *C. crescentus*^*b*^

Protein	*E. coli*	*P. aeruginosa*	*C. crescentus*	Identity		Function
				*E. coli*	*P. aeruginosa*	
Inner membrane						
AmpG	*ampG*	*ampG*	CC_0137	35	37	Muropeptide permease
MurP	*murP*	NF	NF	---	---	Anhydro-N-acetylmuramic acid permease
NagE	*nagE*	PA3761	CC_0538	17	45	N-acetylglucosamine phosphotransferase
Cytoplasm						
NagZ	*nagZ*	PA3005	CC_2006	30	35	β-N-acetyl-D-glucosaminidase
AmpD	*ampD*	PA4522	CC_2567	28	33	N-acetylmuramyl-L-alanine amidase
LdcA	*ldcA*	PA5198	NF	---	---	LD-carboxypeptidase
AnmK	*anmK*	PA0666	CC_1869	34	35	Anhydro-N-acetylmuramic acid kinase
MurQ	*murQ*	NF	NF	---	---	MurNAc-6-P etherase
AmgK	NF	PA0596	CC_3535	---	32	MurNAc kinase
MupP	*mupP*	PA3172	CC_2305	29	29	Phosphoglycolate phosphatase
MurU	NF	PA0597	CC_3536	---	36	N-acetylmuramate alpha-1-phosphate uridylyltransferase
NagA	*nagA*	PA3758	CC_0534	36	34	N-acetylglucosamine-6-phosphate deacetylase
NagB	*nagB*	NF	CC_0535	21	---	Glucosamine-6-phosphate deaminase
MpaA	*mpaA*	PA2831	CC_0311	26	24	Murein peptide amidase A
Mpl^*a*^	*mpl*	*mpl*	*mpl*	53	54.5	Murein peptide ligase

^*a*^ Not found in *C. crescentus* (NA100) strain but in Caulobacteraceae family.

^*b*^ Multiple sequence alignment using the clustalW tool was performed, and percentage identity was estimated between the recycling gene sequences of *E.coli*, *P. aeruginosa,* and *C. crescentus.* NF, not found; ---, these genes are not found in *C. crescentus* genome and hence no identity percentage is represented.

### Cells lacking both SdpA and AmpG display morphological and growth defects

**Fig 9:**
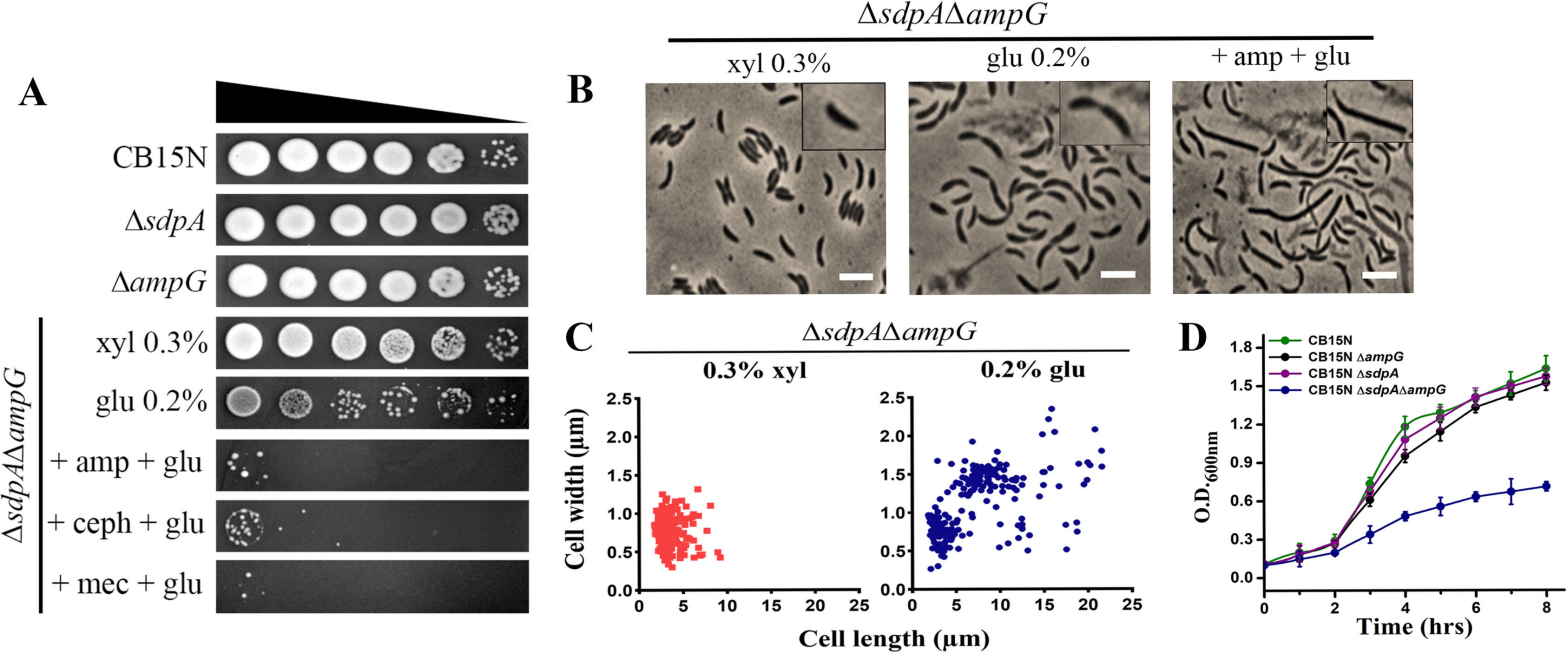
Synthetic effects of AmpG and SdpA deficiency. (**A**) Antibiotic susceptibility was tested by spot dilution assay. Strains CB15N, RP47 (∆*sdpA*), RP56 (∆*ampG*), and RP60 (∆*sdpA*∆*ampG*) strains were serially diluted and spotted on medium (PYE + xylose) or lacking inducer (PYE + glucose) containing antibiotics at different concentrations (ampicillin 10 µg/mL, cephalexin 5 µg/mL, and mecillinam 10 µg/mL). No-antibiotic PYE plates were used as a control. (**B**) Strain RP60 (∆*sdpA*∆*ampG*) was imaged in the presence of 0.2% glucose to deplete *ampG* and glucose + ampicillin (30 µg/mL) with a phase contrast microscope. Cells grown in 0.3% xylose were used as a control (scale bar = 3 µm). (**C**) Cell length analysis of SdpA and AmpG deficient cells. The micrographs described in (**B**) of strain RP60 were analyzed in control (+ xylose) and depletion conditions (+ glucose) at different time points by Oufti Software. The values obtained are shown as scatter plots, plotted as cell length vs cell width (*n* = 215 cells per condition). (**D**) Growth rate analysis of double-deletion mutant RP60 (∆sdpA∆ampG) along with strains CB15N, RP47 (∆*sdpA*), and RP56 (∆*ampG*), indicating reduced viability. Strains were grown in PYE + xylose and PYE + glucose (lacking inducer), and OD_600_ was measured every 2 h. Data represent the mean of three biological replicates.

We further investigated the antibiotic resistance of the *C. crescentus* strain lacking both *sdpA* and *ampG*. To generate this, we introduced a single copy of *ampG* under the control of a xylose-inducible promoter into the chromosome via homologous recombination in the ∆*sdpA* background. Under glucose conditions, ∆*sdpA*∆*ampG* mutant (RP60) suppressed the expression of *ampG*. As expected, the double mutants displayed enhanced sensitivity toward β-lactam antibiotics (Fig. 9A). *C. crescentus* cells lacking either *sdpA* or *ampG* did not exhibit growth defects (Fig. S3). However, phase contrast imaging of double mutants lacking both *sdpA* and *ampG* showed drastic changes in cell morphology (Fig. 9B). While the ∆*sdpA*∆*ampG* mutant displayed regular cell dimensions (*l* = 3.50 ± 0.71 µm, *w* = 0.72 ± 0.09 µm) in the presence of xylose, induction with glucose led to an increase in both cell length (*l* = 7.50 ± 0.21 µm) and width (*w* = 1.20 ± 0.07 µm) (Fig. 9C). The severity of the defect was aggravated in the presence of ampicillin (Fig. 9B). Additionally, the growth curve assay revealed a significant reduction in cell growth for the ∆*sdpA*∆*ampG* double mutant compared to single mutants (Fig. 9D).

### The abundance of PG precursors is reduced in ∆*sdpA* and ∆*ampG* mutants

**Fig 10:**
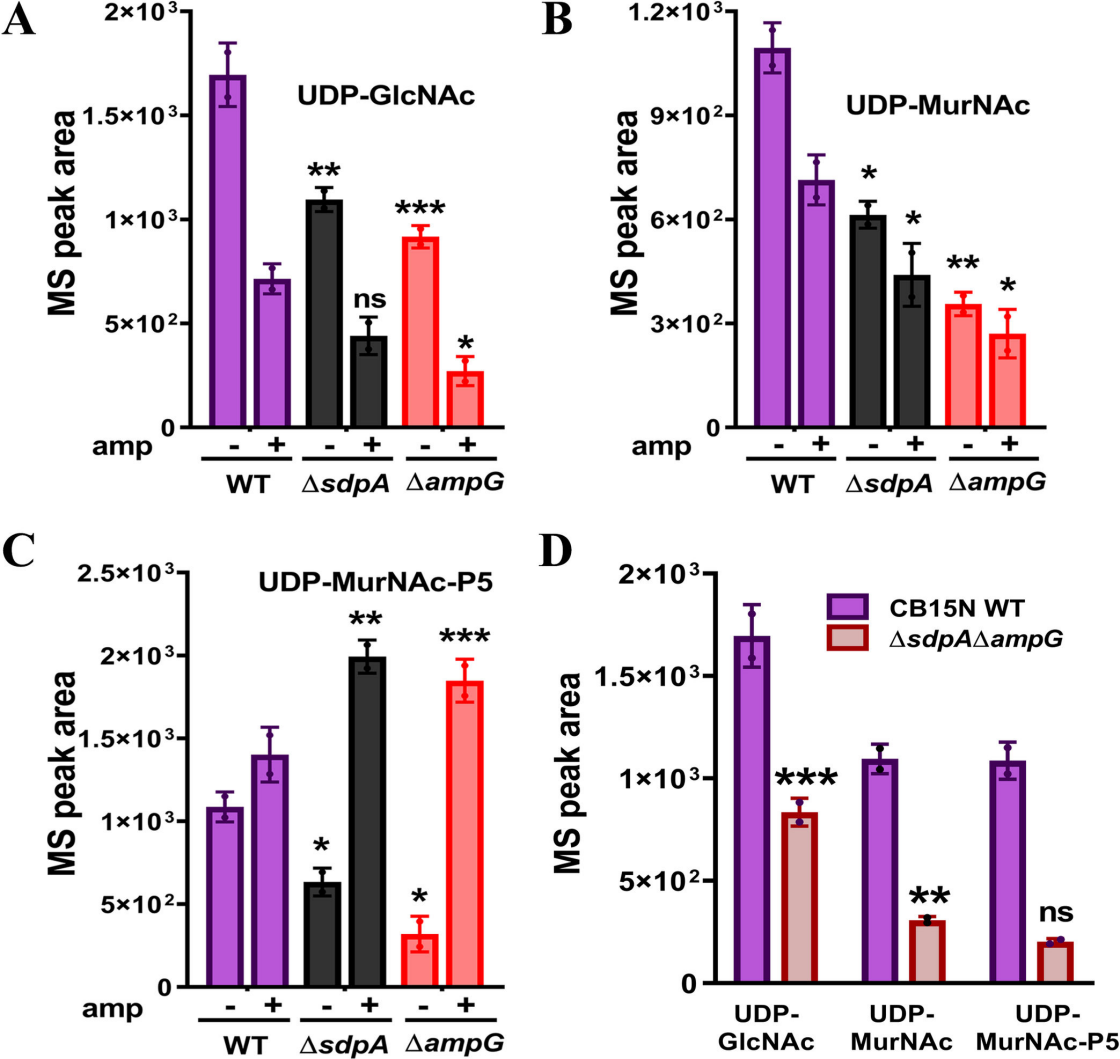
LC-MS analysis of soluble muropeptides in PG recycling mutants. Graphs representing LC-MS quantification of soluble PG muropeptides in the cytoplasmic fraction of strains CB15N (WT), RP47 (∆*sdpA*), and RP56 (∆*ampG*) in the presence (+ amp) and absence of ampicillin detected by LC-MS where panel **A** represents the quantification of PG precursor UDP-GlcNAc (theoretical *m*/*z*, 606.074; observed *m*/*z*, 606.072). Panel **B** represents UDP-MurNAc (theoretical *m*/*z*, 678.095; observed *m*/*z*, 606.092) and panel **C** represents the level of UDP-MurNAc-P5* (theoretical *m*/*z*, 1,193.34; observed *m*/*z*, 595.66) [* = (M – 2H) 2–]. (**D**) Graphs representing LC-MS quantification of soluble PG muropeptides (UDP-GlcNAc, UDP-MurNAc, and UDP-MurNAc-P5) of strain RP60 (∆*sdpA*∆*ampG*) vs WT. Data represent the mean of two biological replicates. Error bars represent the standard deviation (*P < 0.05, **P < 0.005, ***P < 0.005, ****P < 0.0005 by *t*-test , WT vs RP47, RP56, and RP60).

We used LC-MS to analyze and compare the soluble muropeptide composition of the strains CB15N WT, ∆*sdpA*, ∆*ampG,* and double mutants (∆*sdpA*∆*ampG*). Accumulation of various cell wall degradation fragments was detected, including 1,6-anhMurNAc sugar (anhM) along with GlcNAc-anhMurNAc-tri (G-anhM-3), tetra (G-anhM-4), and pentapeptides (G-anhM-5) (Fig. S4A, Table S1). Accumulation of anhydromuropeptides anhM, G-anhM, and G-anhM-5 was significantly reduced in PG recycling single and double mutants in comparison to wild-type strain (Fig. S4B). Essential PG synthesis precursors UDP-GlcNAc, UDP-MurNAc, and UDP-MurNAc-P5 were also decreased in ∆*sdpA*, ∆*ampG,* and double mutants (∆*sdpA*∆*ampG*) compared to wild-type (Fig. 10). We further checked the levels of PG precursors under β-lactam antibiotic stress conditions. The abundance of both UDP-GlcNAc and UDP-MurNAc was further significantly reduced in both ∆*sdpA* and ∆*ampG* mutants (Fig. 10A and B) compared to the wild type in the presence of ampicillin. Conversely, the level of UDP-MurNAc-P5 was increased in the presence of ampicillin in both ∆*sdpA* and ∆*ampG* mutants (Fig. 10C), which is probably due to the loss of transpeptidation activity under β-lactam stress (59).

**Fig 11:**
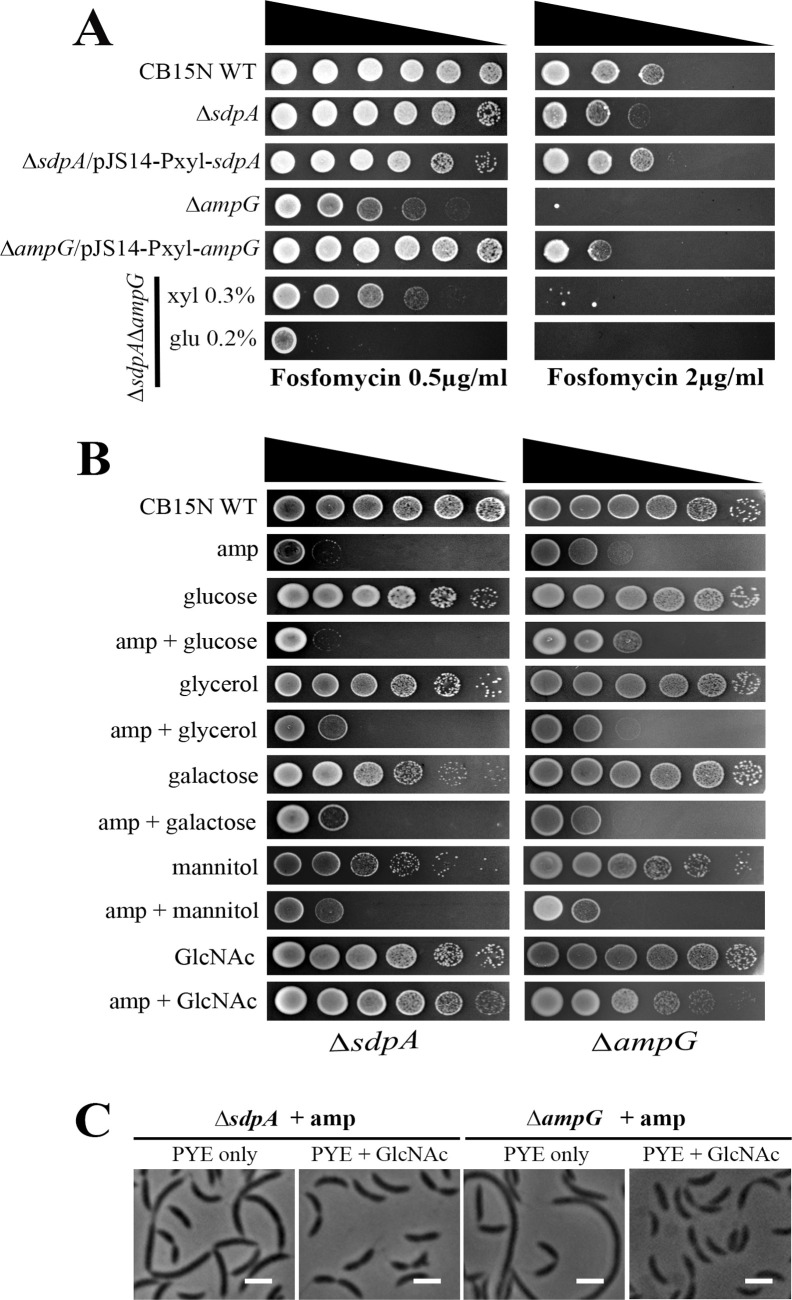
Spot dilution assay in the presence of fosfomycin and different sugar supplements. (**A**) Hypersensitivity of double-deletion knockouts toward fosfomycin (∆*sdpA*∆*ampG*). Spot dilutions were performed for the fosfomycin susceptibility assay. Diluted cultures (6 µL) of strains CB15N, RP47 (∆*sdpA*), RP52 (∆*sdpA*/pJS14/Pxyl/*sdpA*), RP56 (∆*ampG*), RP58 (∆*ampG*/pJS14-Pxyl-*ampG*), and RP60 (∆*sdpA*∆*ampG*) were spotted on PYE + 0.3% xylose and 0.2% glucose containing fosfomycin at two different concentrations (0.5 and 2 µg/mL). All plates were incubated at 30°C for 36 h before imaging. (**B**) Adding GlcNAc reduces the sensitivity of PG recycling mutants toward β-lactam drugs. Diluted cultures (6 µL) of strains CB15N, RP47 (∆*sdpA*), and RP56 (∆*ampG*) were spotted on PYE supplemented with 0.3% glucose, glycerol, galactose, mannitol, and GlcNAc in the presence (+ amp) and absence (– amp) of ampicillin (10 µg/mL). (**C**) Phase contrast images of RP47 (∆*sdpA*) and RP56 (∆*ampG*) cells grown in PYE media supplemented with 0.3% GlcNAc and treated with 40 µg/mL ampicillin for 6 h at 30°C (scale bar = 2 µm).

One plausible explanation for reduced levels of PG precursor is that PG recycling products contribute to PG precursor synthesis in the cytoplasm. If there is a coupling of PG recycling and *de novo* PG synthesis pathways in *C. crescentus*, then PG recycling mutants would fare poorly to any perturbation in *de novo* PG synthesis. A similar scenario has been proposed in *A. tumefaciens* (51) and *P. aeruginosa* (60). We subjected both *sdpA* and *ampG* mutants to fosfomycin treatment to investigate this hypothesis. Fosfomycin inhibits MurA (61) and as predicted, double knockout mutants (∆*sdpA*∆*ampG*) were hypersensitive to fosfomycin (Fig. 11A). Additionally, Δ*ampG* cells exhibited enhanced sensitivity toward fosfomycin in comparison to Δ*sdpA* (Fig. 11A). As the abundance of both PG amino-sugar precursors UDP-GlcNAc and UDP-MurNAc was decreased in the ∆*sdpA* and ∆*ampG* single and double mutants compared to wild type (Fig. 10A through C), we postulated that supplementing the growth media with GlcNAc may increase the fitness of ∆*sdpA* and ∆*ampG* mutants in antibiotic stress. Both ∆*sdpA* and ∆*ampG* mutants displayed increased resistance to ampicillin treatment in peptone-yeast extract (PYE) medium supplemented with 0.3% GlcNAc (Fig. 11B). Furthermore, the fitness of ∆*sdpA*∆*ampG* mutants was enhanced in PYE media supplemented with GlcNAc (Fig. S5). To further confirm that increased fitness in GlcNAc-supplemented media was not due to the addition of sugar, a similar experiment was performed by supplementing the media with glucose, galactose, mannitol, or glycerol (Fig. 11B). None of the other sugar supplements were able to increase the fitness of *sdpA* or *ampG* mutants upon antibiotic challenge (Fig. 11B). To further confirm the postulated hypothesies, supplementation of GlcNAc in PYE growth medium prior to antibiotic treatment should restore normal cell morphology. To confirm this, ∆*sdpA* and ∆*ampG* cells were grown in the presence of GlcNAc and subjected to ampicillin treatment. GlcNAc supplementation rescued the morphological defects of ∆*sdpA* and ∆*ampG* on ampicillin treatment (Fig. 11C). Taken together, our results suggest that PG turnover products are utilized in the PG synthesis pathway in *C. crescentus*.

### Peptidoglycan amino sugar recycling genes are found in *C. crescentus*

**Fig 12:**
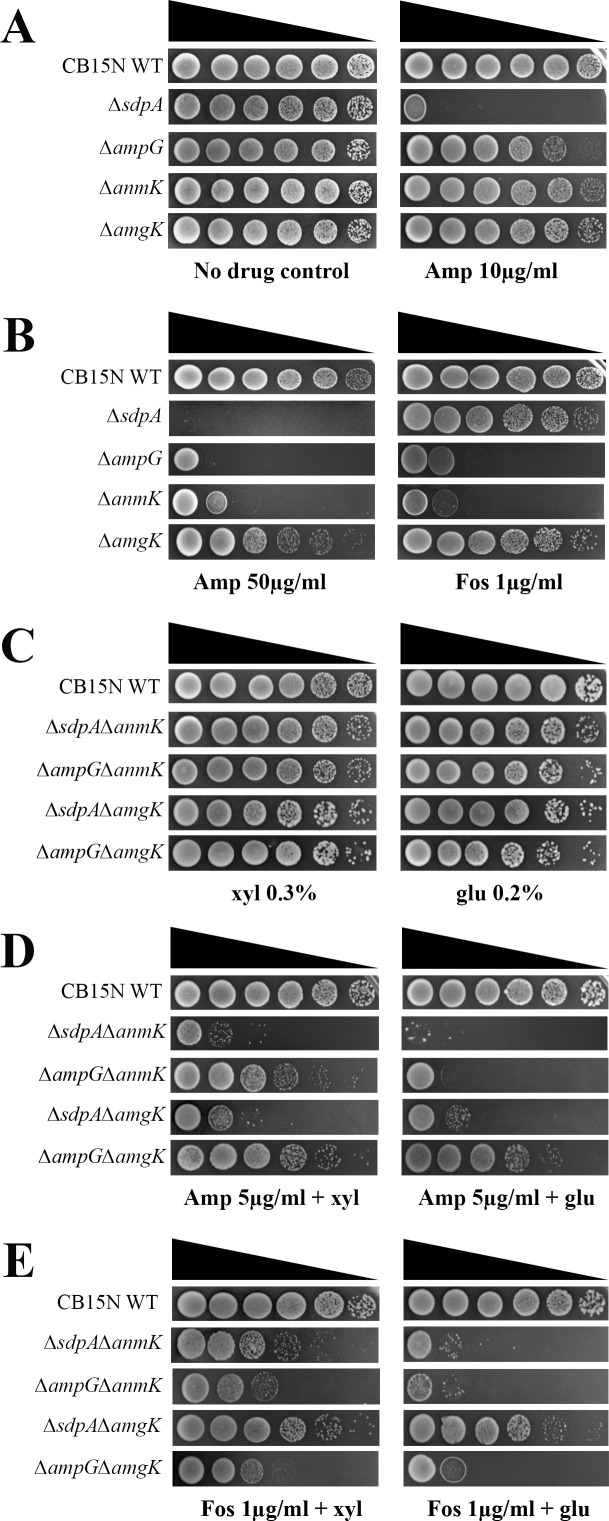
(**A, B**) Antibiotic sensitivity of ∆*anmK* and ∆*amgK* PG recycling mutants. Cultures of strains WT, RP47 (Δ*sdpA*), RP56 (Δ*ampG*), RP64 (Δ*anmK*), and RP65 (Δ*amgK*) were serially diluted, and 5 µL of each dilution was spotted onto the PYE agar supplemented with either 50 µg/mL ampicillin or 1 µg/mL fosfomycin. PYE agar media without antibiotics were used as no-drug control. (**C–E**) Cultures of strains WT, RP68 (Δ*sdpA*Δ*anmK*), RP70 (Δ*ampG*Δ*anmK*), RP69 (Δ*sdpA*Δ*amgK*), and RP71 (*ΔampGΔamgK*) were serially diluted, and about 5 µL of each dilution was spotted on medium (PYE + xylose) or lacking inducer (PYE + glucose) supplemented with ampicillin (5 µg/mL) and fosfomycin (1 µg/mL).

Recycling of peptidoglycan amino sugars also provides precursors for the PG synthesis pathway. Two different pathways are known to process the sugar residues of anhydromuropeptides. In *E. coli*, catabolic activities of MurQ and NagA modify MurNAc saccharide to glucosamine-6-phosphate for use in the *de novo* PG biosynthesis pathway (20, 62). Some *Pseudomonas* family members lack MurQ and NagK, and utilize an anabolic route to recycle the sugar moieties (63). AnmK phosphorylates anhMurNAc and generates MurNAc-6-P, which is further processed into the PG recycling pathway (20). MupP converts MurNAc-6-P to MurNAc, which is then acted on by AmgK kinase to produce MurNAc-1-P (60). MurU uses this MurNAc-1-P to give UDP-MurNAc, which is channeled to the *de novo* PG biosynthesis pathway (64, 65). Homologs for NagA (*CC_0534*), NagB (*CC_0311*), MupP (*CC_2305*), AmgK (*CC_3535*), and AnmK (*CC_1869)* (Table 1) are encoded by the *C. crescentus* genome. To further investigate the utilization of peptidoglycan amino sugars in PG synthesis, we generated single deletions of *anmk* (RP64) and *amgK* (RP65), and tested their fitness under antibiotic stress. The *amgK* mutant displayed sensitivity to ampicillin concentrations of 50 µg/mL or higher and to fosfomycin at 1 µg/mL (Fig. 12A). In contrast, the *anmK* mutant cells were sensitive to lower antibiotic concentrations similar to those observed in *sdpA* and *ampG* mutants (Fig. 12A). Furthermore, we combined the single mutants of *sdpA* and *ampG* with either *anmk* or *amgK* to create double-deletion mutants: ∆*sdpA*∆*anmK* (RP68), ∆*sdpA*∆*amgK* (RP69), ∆*ampG*∆*anmK* (RP70), and ∆*ampG*∆*amgK* (RP71). In these strains, the expression of both *anmK* and *amgK* is under the control of the xylose promoter. In glucose conditions, the growth of these mutants results in the repression of *amgK* or *anmK* expression, leading to a double-deletion background. All double mutants showed compromised fitness at lower concentrations of ampicillin (5 µg/mL) (Fig. 12B). All double knockout mutants were extremely sensitive to fosfomycin compared to wild type (Fig. 12B), suggesting that the absence of amino sugar recycling in these mutants results in the decreased pool of PG precursors, thereby increasing antibiotic sensitivity. These results suggest that *C. crescentus* probably utilizes amino sugars generated from PG degradation into the PG synthesis pathway.

## DISCUSSION

PG recycling has gained importance due to its role in antibiotic resistance among many gram-negative bacteria. However, PG recycling pathways have been delineated only in a few model organisms and, to date, remain poorly understood in a broad range of bacterial species. *C. crescentus* contains nearly all the genes necessary for PG recycling, with the exceptions of LdcA, MurQ, and Mpl (Table 1). Analysis revealed the presence of various muropeptides, including GlcNAc-anhMurNAc-tri, tetra, and pentapeptides, in *C. crescentus* cells, indicating the PG turnover process. Anhydromuropeptides accumulated in the periplasm in the absence of AmpG, indicating that AmpG is essential for the transport of peptidoglycan fragments generated by the action of lytic transglycosylases. In the cytoplasm, muropeptides are further processed by LdcA, NagZ, and AmpD. The tripeptide produced by LdcA is combined with UDP-MurNAc, contributing to the pool of UDP-MurNAc-tripeptide available for *de novo* peptidoglycan biosynthesis (66). *C. crescentus* has homologs for NagZ (*CC_2006*) and AmpD (*CC_2567)* (Table 1) but lacks the homolog for LdcA (Table 1). Although Mpl homologs are found in other members of the *Caulobacteraceae* family, none were identified in the *C. crescentus* genome. It is plausible that *C. crescentus* does not recycle the peptides but instead degrades them into amino acids for utilization in other metabolic pathways. MpaA (*CC_0311*), a murein tripeptide amidase that cleaves the γ-D-glutamyl-*meso*-diaminopimelic acid bond in the murein tripeptide (L-Ala-γ-D-Glu-*m*DAP), is present in *C. crescentus* (Table 1).

*C. crescentus* recycles peptidoglycan amino sugars, and we have identified signatures of both catabolic and anabolic PG recycling pathways in its genome. The genome of *C. crescentus* encodes homologs for NagA, NagB, MupP, AmgK, and AnmK (Table 1). However, BLAST searches for MurQ did not yield significant hits. Recycled amino sugar products are further utilized in the synthesis of murein precursors. Deletion of AnmK resulted in increased sensitivity to fosfomycin, and mutants lacking both AnmK and AmpG were fitness compromised at low β-lactam concentrations (Fig. 12). In contrast, the deletion of AmgK did not significantly affect β-lactam resistances (Fig. 12), probably due to the presence of a compensatory catabolic pathway. Because *C. crescentus* typically inhabits oligotrophic environments, recycling PG degradation products would be beneficial for the bacterium.

PG recycling genes *ampG* and *sdpA* are not essential under normal laboratory growth. Moreover, drastic changes were not observed in the cell wall from *sdpA* and *ampG* mutants (Table S3). Interestingly, *Caulobacter* cells lacking both *sdpA* and *ampG (*∆*sdpA*∆*ampG*) displayed severe growth and morphological defects, indicating that PG recycling probably plays a role in maintaining cell wall integrity (Fig. 7). Quantitative analysis of the soluble cytoplasmic PG precursors showed a significant decrease in UDP-GlcNAc, UDP-MurNAc, and UDP-MurNAc-P5. PG precursor level further declined in ∆*sdpA*∆*ampG* mutants, indicating that PG recycling products serve as an essential source for *de novo* PG biosynthesis (Figure 10D). These results were further corroborated by fosfomycin hypersensitivity in both ∆*ampG* and ∆*sdpA*∆*ampG* mutants (Fig. 11A). A similar scenario has been observed in *A. tumefaciens*, where PG recycling contributes materials for PG synthesis. Moreover*,* the deletion of the PG recycling transporter, yejABEF, leads to growth defects, altered cell wall composition, and hypersensitivity to β-lactam antibiotics in *A. tumefaciens*.

*C. crescentus* cell wall has a high abundance of 1-6-anhydromuropeptides (67), which is believed to be due to the high activity of LTs. *C. crescentus* encodes three SLTs—SdpA/B/C (41). Despite having functional redundancy, only SdpA is essential for β-lactam resistance (Fig. 1A) (41). Similarly, in *P. aeruginosa* and *A. tumefaciens,* only a single LT is essential for β-lactam resistance (30, 44). In the case of *A. tumefaciens*, the loss of a single membrane-bound lytic transglycosylase leads to ampicillin hypersensitivity (29). *C. crescentus* is intrinsically resistant to β-lactams due to the activity of potent β-lactamase (Mbl1B) (54). Interestingly, deletions of LTs did not affect the expression and activity of Mbl1B. Moreover, β-lactamase activity was not diminished in the *ampG* mutant (Fig. 7A and B), suggesting that *sdpA* and *ampG* may not play a role in the regulation of intrinsic β-lactamase in *C. crescentus*.

What could be the reason for sensitivity in ∆*sdpA* and ∆*ampG* mutants? One plausible explanation could be that the defects in PG recycling could distress the *de novo* PG synthesis pathway. Both UDP-MurNAc and UDP-GlcNAc levels are decreased upon antibiotic treatment, and growth media supplementation with GlcNAc was able to rescue the antibiotic sensitivity in Δ*sdpA* and Δ*ampG* mutants (Fig. 11), suggesting that *C. crescentus* may recycle sugar moieties to generate PG monomers. Moreover, the growth rate and viability of ∆*sdpA*∆*ampG* mutant were rescued in media supplemented with GlcNAc sugar (Fig. S5), which enters inside the cytoplasm via the NagE phosphotransferase system (Fig. 8, Table 1) and is further processed into murein precursors. Collectively, these results suggest a direct link between PG recycling and PG synthesis pathways in *C. crescentus*.

We further examined SdpA in the Fitness Browser database (68) under β-lactam antibiotic stress. The cofitness data revealed strong correlations between SdpA and AmpG under β-lactam pressure. Cofitness data also predicted a strong relation between SdpA and membrane-bound lytic murein transglycosylase B (MltB), and the role of MltB in β-lactam stress should be explored. *C. crescentus* elongates from the mid-cell by FtsZ ring-mediated medial accumulation of MurG (69). SdpA is also recruited to mid-cell in an FtsZ-dependent manner (41). This might help the cells to efficiently coordinate between PG synthesis and PG degradation activities. The overexpression of SdpA leads to defects in cell division and cell viability, suggesting a compromised cell wall integrity. During cell growth, cell wall remodeling enzymes release muropeptides that are transported into the cytoplasm. These degradation products directly contribute toward synthesizing new cell wall precursors, which are then transported to the periplasm and eventually incorporated into the cell wall. Deficiency in PG recycling probably causes a reduction in cell wall biogenesis, which is deleterious under antibiotic stress.

## MATERIALS AND METHODS

### Media and growth conditions

*C. crescentus* strains were acquired from synchronizable wild-type strain CB15N (NA1000) and grown in PYE media supplemented with 1 M MgSO_4_ and 1 M CaCl_2_ or M2G minimal media at 30°C (45). Xylose (0.3%) and glucose (0.2%) were used for induction and repression of *xylX* promoter at 0.1 OD_600_ unless stated otherwise. Induction of the *vanA* promoter was carried out by 0.5 mM vanillic acid. For *C. crescentus*, antibiotics were used in the following concentrations unless stated otherwise—kanamycin (50 µg/mL), gentamycin (5 µg/mL), streptomycin (50 µg/mL), chloramphenicol (2 µg/mL), oxytetracycline (5 µg/mL), and nalidixic acid (20 µg/mL) for growth. *E. coli* strains were cultured in Luria-Bertani broth (LB media) at 37°C supplemented with antibiotics in concentrations—ampicillin (100 µg/mL), kanamycin (50 µg/mL), streptomycin (50 µg/mL), chloramphenicol (30 µg/mL), and oxytetracycline (10 µg/mL) for growth. Antibiotics used in this study were purchased from Sigma-Aldrich (Milwaukee, WI, USA), and media were purchased from Himedia (Mumbai, India). The strains and plasmids used in this study are listed in Table 2, and their construction is mentioned in the supplemental text.

**TABLE 2:** List of strains and plasmids used in this study

Plasmids	Relevant genotype or description	Sources/references
pJS14	High copy number vector, pBR1MCS derivative, Cmr	Laboratory strain collection
pNTPS138	Kan^r^, suicide vector with SacB gene and oriT	Laboratory strain collection
pXMCS-5	Integration vectors for the one-step generation of depletion strains, Tet^r^	(70)
pMCS-1	Vectors for integration at a site of interest, Strep^r^	(70)
pXGFPN-2	Vector used for generating N-terminal protein fusions encoded at xylX locus, Kan^r^	(70)
pVYFPN-4	Vector used for generating N-terminal protein fusions encoded at vanA locus, Gent^r^	(70)
pMM1	pNTPS138-based plasmid for constructing an in-frame deletion in *sdpA*	This work
pMM2	pJS14 carrying *sdpA* under xylose promoter	This work
pMM3	pJS14 carrying *sdpB* under xylose promoter	This work
pMM4	pJS14 carrying *sdpC* under xylose promoter	This work
pMM5	pJS14 carrying *sdpA (Q544E)* (active site mutant) under xylose promoter	This work
pMM6	pNTPS138-based plasmid for constructing an in-frame deletion in *CCNA_00136*	This work
pMM7	pJS14 carrying *CCNA_00136* under xylose promoter	This work
pMM8	pXMCS-5 carrying *CCNA_00136_1-203aa_*	This work
pMM9	pJS14 carrying *CCNA_02650* under xylose promoter	This work
pMM10	pMCS-1 carrying *CCNA_02650_1-242aa_*	This work
pMM11	pXGFPN-2 carrying *CCNA_00136*	(71)
pMM12	pMCS-1 carrying *CCNA_01945_1-300a_*	This work
pMM13	pMCS-1 carrying *CCNA_03649_1-382aa_*	This work
pMM14	pXMCS-5 carrying *CCNA_01945_1-300a_*	This work
pMM15	pXMCS-5 carrying *CCNA_03649_1-382aa_*	This work
DH5α	Cloning strain Φ80 ΔlacZΔM15 Δ(lacZYA-argF)U169 deoR recA1 endA hsdR17(rk-,mk+) phoA supE44 thi-1 gyrA96 relA1	Laboratory strain collection
S17	RP4-2, Tc::Mu, KM-Tn7	(72)
CB15N	Synchronizable derivative of wild-type strain CB15	(73)
RP2	CB15N *cc2007*::pGVan2007 YFP xylX::pXCHYC-2-*amiC*	(71)
RP3	CB15N *ftsZ*::pBJM1 *vanA*::pVCHYC-2-*amiC*	(71)
RP47	CB15N ∆*sdpA*::Ω	This work
RP48	CB15N/pJS14-P*xyl-sdpA*	This work
RP49	CB15N/pJS14-P*xyl-sdpB*	This work
RP50	CB15N/pJS14-P*xyl-sdpC*	This work
RP51	CB15N/pJS14-P*xyl-sdpA*(Q544E)	This work
RP52	CB15N ∆*sdpA*/pJS14-P*xyl-sdpA*	This work
RP53	CB15N ∆*sdpA*/pJS14-P*xyl-sdpB*	This work
RP54	CB15N ∆*sdpA*/pJS14-P*xyl-sdpC*	This work
RP55	CB15N ∆*sdpA*/pJS14-P*xyl-sdpA*(Q544E)	This work
RP56	CB15N ∆*ampG*::Ω	This work
RP57	CB15N/pJS14-P*xyl-ampG*	This work
RP58	CB15N ∆*ampG/*pJS14-P*xyl-ampG*	This work
RP59	CB15N P*xyl*::P*xyl*-pXMCS-5-*ampG_1_*_-203aa_	This work
RP60	CB15N ∆*sdpA* P*xyl*::P*xyl*-pXMCS-5-*ampG_1_*_-203aa_	This work
RP61	CB15N P*van*::P*van-ftsN-yfp/*pJS14-P*xyl-sdpA*	This work
RP62	CB15N P*van*::P*van-ftsZ-yfp*/pJS14-Pxyl-*sdpA*	This work
RP63	CB15N pMCS-1-*ampD_1-203aa_*	This work
RP64	CB15N pMCS-1-*anmK_1-300aa_*	This work
RP65	CB15N pMCS-1-*amgK_1-382aa_*	This work
RP66	CB15N P*xyl*::P*xyl*-pXMCS-5-*anmK_1_*_-*300aa*_	This work
RP67	CB15N P*xyl*::P*xyl*-pXMCS-5-*amgK_1_*_-*382aa*_	This work
RP68	CB15N ∆*sdpA* P*xyl*::P*xyl*- pXMCS-5-*anmK_1-300a_*	This work
RP69	CB15N ∆*sdpA* P*xyl*::P*xyl*- pXMCS-5-*amgK_1-382a_*	This work
RP70	CB15N ∆*ampG* P*xyl*::P*xyl*- pXMCS-5-*anmK_1-300a_*	This work
RP71	CB15N ∆*ampG* P*xyl*::P*xyl*- pXMCS-5-*amgK_1-382a_*	This work
RP72	CB15N P*xyl*::P*xyl*-GFP*-ampG*	This work

### Synchronization of GFP-AmpG fusion construct

Synchronization of *C. crescentus* cells was performed using mini-synchrony protocol. Cells were grown in PYE media (200 mL) up to exponential phase (OD_600_ 0.4) and pelleted at 5,000 rpm for 15 min at 4°C. The cell pellet was resuspended in 30 mL ice-cold M2 media and mixed with 10 mL ice-cold Ludox (Sigma-Aldrich). The resuspended solution was centrifuged at 9,000 rpm for 40 min at 4°C, and the swarmer cells was aspirated. Swarmer cells were washed thrice with cold M2 media, and the final pellet was resuspended in prewarmed PYE media supplemented with 0.1% xylose. At every 15 min interval, about 500 µL cells were pelleted down and imaged.

### Immunoblot analysis

The stability of the GFP-AmpG fusion construct was confirmed by Western blotting with an anti-GFP antibody (Invitrogen, Waltham, MA, USA). Strain RP 72 (CB15N P*xyl::*P*xyl-*GFP*-ampG*) was grown until OD_600_ ∼0.1 and was induced with 0.2% glucose or 0.3% xylose. After induction, the cells were grown for an additional 3 h before being subjected to Western blot analysis. Immunocomplex was detected using Bio-Rad Clarity and clarity Max ECL Western Blotting Substrates (Bio-Rad Laboratories, Inc., USA) according to the manufacturer’s protocols.

### Microscopy and image analysis

Light microscopy imaging was performed using 100× objectives of the differential interference microscope and phase contrast microscope on a Nikon Eclipse Ts2R microscope equipped with a Nikon DS-Fi3 camera and Nikon Plan Fluor 100X oil Ph3 objective. Cells were immobilized on a 1% agarose pad in M2G media for DIC and phase contrast time course experiments (71). For DIC time-lapse imaging, PYE agarose pads were supplemented with either 0.3% xylose or 0.2% glucose for induction or repression of the *xylA* promoter.

Fluorescence imaging was conducted using the Nikon Eclipse Ti Microscope equipped with Nikon Plan Apo 100X (NA1.40) oil objective and a Nikon DS-U3 camera. For visualization of inducible fluorescent fusion proteins, cells were grown to exponential phase in PYE media and were induced with 0.5 mM vanillic acid or 0.3% xylose, or both at 0.1 OD_600_. Single-image acquisitions were made every 3 h. A time-lapse movie representing the localization of FtsN-YFP fusion protein was recorded in Nikon A1R MP+Ti-E confocal microscope system provided with solid-state lasers on M2 media 1% agarose pads supplemented with inducer. Image processing was performed in Adobe Photoshop CS6. Automated image analysis was carried out with MATLAB-dependent Oufti Software (74). Cell length analysis graphs were plotted on Origin Software (Origin 7.5, OriginLab Corp., Northampton, MA, USA).

### Site-directed mutagenesis

Active-site residue of *C. crescentus sdpA* gene was identified by multiple sequence alignment using MultAlin (Florence Corpet) (75) tool, with *E. coli* Slt70, which shows 32% identity between both sequences (Fig. S1). The QuickChange Method developed by Stratagene (La Jolla, CA, USA) was used to perform site-directed mutagenesis (76). The SdpA active-site mutant was generated by introducing a point mutation at position 544 by replacing glutamine at the active site with glutamic acid. Plasmid pJS14/Pxyl-*sdpA* was amplified using primers FP (5′-ATCTCGCGGCAGCAGAGCAATTTC-3′) and RP (5′-GTCGAAATTGCTCTGCTGCCGCGA-3′). The parental plasmid was digested with the Dpn1 enzyme and transformed into DH5α, followed by antibiotic selection. Mutant clones were confirmed by sequencing.

### Spot dilution assay experiment

For analyzing the viability of *C. crescentus* strains, different β-lactam antibiotics were used in varied concentrations—ampicillin (10 and 30 µg/mL), cephalexin (5 and 8 µg/mL), mecillinam (10 and 20 µg/mL), and non-β-lactam drugs like vancomycin (20 µg/mL) and fosfomycin (0.5–2 µg/mL); all wild-type and mutant strains were grown overnight in PYE media. Cells were then diluted to 0.05 OD_600_ and were induced at 0.1 OD_600_ as required. Cells were grown until the mid-exponential phase before being normalized to the optical density of 0.1 and 10-fold serially diluted. About 10 µL of each serial dilution was spotted on PYE + β-lactam drug plate supplemented with 0.3% xylose, if necessary. Plates were incubated at 30°C for 48 h before imaging.

### HADA labeling

HADA hydrochloride 3-[[(7-hydroxy-2-oxo-2H-1-benzopyran-3-yl)carbonyl]amino]-D-alanine hydrochloride (MedChemExpress, Cat. #HY-131045/CS-0124027), a fluorescent D-amino acid (FDAA), can efficiently incorporate into the PG at the site of PG biosynthesis. Cells were labeled with HADA as described previously (77). Strains CB15N, RP47 (∆*sdpA*), and RP56 (∆*ampG*) were treated with ampicillin (40 µg/mL) at 0.1 O.D_600_ and grown for 3 h. Cells were collected and washed with M2 media twice. HADA was added to 0.5 mM and incubated at 30°C for 15 min. Cells were again washed with M2 media and resuspended in M2 media before imaging.

### Nitrocefin hydrolysis test

β-Lactamase activity of PG recycling mutants was measured by Nitrocefin (purchased from MedChemExpress, USA), a chromogenic substrate as per protocol described earlier (36) with slight modifications. Strains CB15N, RP47 (∆*sdpA*), and RP56 (∆*ampG*) were grown until OD_600_ ∼0.1, treated with 40 µg/mL ampicillin, and incubated for 4 h at 30°C. About 200 µL of both treated and untreated samples was aliquoted, and the cell concentrations were normalized. Samples were mixed with 1 mg/mL Nitrocefin, and absorbance at 490 nm (A490) was measured in a TECAN Spark plate reader in Kinetic mode at 30°C for 2 h.

### Preparation of soluble and periplast fractions of *C. crescentus*

To analyze soluble PG metabolites, *C. crescentus* CB15N and various mutant strains were grown in 200 mL of PYE media until 1.0 OD_600_. Cell extracts were prepared as described earlier (63). Cells were pelleted down at 7,000 rpm for 15 min and resuspended in Tris-HCl buffer (pH 7.6). The pellet was washed twice with 10 mL Tris-HCl buffer, resuspended in 2 mL of water, and boiled at 95°C for 30 min. The boiled suspensions were harvested at maximum speed for 20 min to collect the soluble cell extracts containing cell wall turnover products. About four volumes of acetone were added to the extract and incubated at −20°C for 36 h. The insoluble part was removed by centrifugation at 10,000 rpm for 15 min. The supernatant was collected and dried at 60°C in a vacuum concentrator. The dried pellet was then dissolved in 400 µL of water.

Cell fractionation was performed to obtain the periplast fraction of PG recycling mutants using the method previously described (35) with slight modifications. One liter culture of strains CB15N, RP47 (∆*sdpA*), and RP56 (∆*ampG*) was grown until mid-exponential phase (OD_600_ ∼0.6) in PYE. The pellet was resuspended in 10 mL of periplasting buffer (50 mM Tris-HCl, pH 8.0, 12% sucrose, and 1 mM CaCl_2_). Lysozyme at 2 mg/mL and EDTA at 1 mM concentration were added, and the suspension was incubated on ice for 45 min. The suspension was pelleted down at 3,500 rpm for 10 min, and the supernatant comprising the periplast fraction of cells was saved in a fresh tube. The periplast fraction was filtered through a 0.22 µm filter and then fractionated in a 10 kDa cutoff concentrator (Millipore, Amicon). The flow-through fraction was collected and subjected to muramidase digestion at 1 mg/mL concentration for 30 min at 4°C. The fraction was concentrated under vacuum and boiled for 10 min at 100°C. The final periplast was stored at 4°C.

### LCMS analysis

Mass spectrometry to analyze soluble and periplast metabolites was performed using a 6540 UHD Accurate-Mass Q-TOF LC/MS system (Agilent Technologies, Santa Clara, CA, USA) equipped with Agilent 1290 UPLC system. For analysis of soluble metabolites inside the cell, about 7 µL of sample injection volume was applied to the Gemini C18 column (Phenomenex, 150 × 4.6 mm, 110 Å, 5 µM) at a flow rate of 0.1 mL/min. A 45 min program in negative ion mode was run involving 5 min of washing with buffer A (0.1% formic acid and 0.05% ammonium formate) and 30 min of a linear gradient to 40% buffer B (100% acetonitrile) along with 5 min of 40% of buffer B and 10 min of re-equilibration step. For periplast fraction LC-MS analysis, a 10 µL sample injection volume was applied at a flow rate of 0.2 mL/min. Analyte separation started with a 5 min wash using buffer A (0.1% formic acid), succeeded by a 20 min linear gradient reaching 100% buffer B (100% acetonitrile), followed by another 5 min wash with buffer A.

LCMS data analysis was carried out in MassHunter Workstation Software – Qualitative Analysis Version B.06.00. Each mutant strain’s abundance value for respective metabolites was then exported to GraphPad Software for further data analysis.

## SUPPLEMENTAL MATERIAL

Supplemental material mbio.02975-24-s0001.docx Supplemental methods, figures, and table.

Movie S1 mbio.02975-24-s0002.mp4 CB115N treated with ampicillin.

Movie S2 mbio.02975-24-s0003.mp4 *sdpA* deletion mutant treated with ampicillin.

Movie S3 mbio.02975-24-s0004.mp4 CB15N treated with cephalexin.

Movie S4 mbio.02975-24-s0005.mp4 *sdpA* deletion mutant treated with cephalexin.

Movie S5 mbio.02975-24-s0006.mp4 CB15N treated with mecillinam.

Movie S6 mbio.02975-24-s0007.mp4 *sdpA* deletion mutant treated with mecillinam.

ASM does not own the copyrights to Supplemental Material that may be linked to, or accessed through, an article. The authors have granted ASM a non-exclusive, world-wide license to publish the Supplemental Material files. Please contact the corresponding author directly for reuse.
