# Supplementary material for: Deficiency in peptidoglycan recycling promotes β-lactam sensitivity in Caulobacter crescentus
Source: mBio. 2025 Mar 11;16(4):e02975-24. doi: 10.1128/mbio.02975-24 (PMC11980594; doi:10.1128/mbio.02975-24)
Supplement: Supplemental material — Supplemental methods, figures, and table. [file mbio.02975-24-s0001.docx]

**Deficiency in peptidoglycan recycling promotes β-lactam sensitivity in *Caulobacter crescentus***

**Malvika Modi^1^, Deepika Chauhan^1¶^, Michael C. Gilmore^2^, Felipe Cava^2^ and Richa Priyadarshini^1*^**

^1^ Department of Life Sciences, School of Natural Sciences, Shiv Nadar University, Gautam Buddha Nagar, Uttar Pradesh, India

^2^Laboratory for Molecular Infection Medicine Sweden, Department of Molecular Biology, Umeå Centre for Microbial Research, Umeå University, Umeå, Sweden

***** Corresponding author

**Correspondence and reprints**:

**Dr. Richa Priyadarshini**

Department of Life Sciences, School of Natural Sciences,

Shiv Nadar Institute of Eminence

Gautam Buddha Nagar, Uttar Pradesh, India.

Ph no: +91-120-3819100 Ext. 220

Email: [richa.priyadarshini@snu.edu.in](mailto:richa.priyadarshini@snu.edu.in) (RP)

**¶ Present Address:**

Bond Life Sciences Center,

University of Missouri, Columbia, MO, USA

**Plasmid Construction**

**pMM1** – Plasmid generated by PCR amplification of the upstream region of CCNA_01252 *sdpA* gene using SdpA F.P 1 – CCCCCAAGCTTGTGCACAGGCCCAGTTGCGC and SdpA R.P 1 – CCCCCGGATCCCTGAAACCAAAACGCTCTCC primers and downstream region of *sdpA* gene using primers SdpA F.P 2 – CCCCCGGATCCGCCTCAACGGCGGCGTCACG and SdpA R.P 2 – CCCCCGCTAGCCTGTCGGTCTCGGCCGGGG. The amplified fragments were ligated into the pNTPS138 vector at HindIII and NheI sites with the BamHI site in between, where the omega cassette was inserted. The construct was further confirmed by sequencing.

**pMM2** – Plasmid generated by PCR amplification of CCNA_01252 (*sdpA*) from CB15N genomic DNA using primers F.P - CCCCATATGTTGGTTTCAGGAATGCGTCG and R.P – CCCCGGTACCTTAAGGCTGGGCGCTGGC, digested with Nde1 and Kpn1 and ligated into digested vector pJS14.

**pMM3** – Plasmid generated by PCR amplification of CCNA_01393 (*sdpB*) from CB15N genomic DNA using primers F.P – CCCCATATGTTGCTTTTGCAGCTGTGGGC and R.P – CCCCGGTACCCTAGGGCAGCTGCGCCATCA, digested with Nde1 and Kpn1 and ligated into digested vector pJS14.

**pMM4** – Plasmid generated by PCR amplification of CCNA_02498 (*sdpC*) from CB15N genomic DNA using primers F.P – CCCCATATGATGGAGGACACACTGGGG and R.P – CCCCGGTACCTCAGGGCTTGCGGCGCGG, digested with Nde1 and Kpn1 and ligated into digested vector pJS14.

**pMM5** – Site-directed mutagenesis was performed with primers F.P – ATCTCGCGGCAGCAGAGCAATTTC and R.P – GTCGAAATTGCTCTGCTGCCGCGA using pMM2 as a template. The resulting mutant plasmid product was digested with the restriction enzyme Dpn1 and transformed into DH5α, and transformants were screened on LB chloramphenicol (30µg/ml) plates. Active site mutant clones were confirmed by sequencing. The resulting plasmid was then transformed into S17 and was further mated with CB15N NA100.

**pMM6** – Plasmid generated by PCR amplification of the upstream region of *ampG* gene using AmpG F.P 1 – CCCCCAAGCTTCGGTGGTGCTGCTGACGCC and AmpG R.P 1 – CCCCCGGATCCTCGCCGAAGAAGGCGAGGTCTCG primers and downstream region of *ampG* gene using primers AmpG F.P 2 – CCCCCGGATCCTCGTCGCGATCGTCCTGTCC and AmpG R.P 2 – CCCCCGAATTCGACCGCCCGCCATGTGGTCG. The amplified fragments were ligated into the pNTPS138 vector at HindIII and EcoRI sites with the BamHI site in between, at which the omega cassette was inserted. The construct was further confirmed by sequencing.

**pMM7** – Plasmid generated by PCR amplification of CCNA_00136 *(ampG)* from CB15N genomic DNA using primers F.P – CCCCATATGATGTCCGAAGAAGCCAAG and R.P – CCCCGGTACCTTAGGAGGCGGCGAGGGT, digested with Nde1 and Kpn1 and ligated into digested vector pJS14.

**pMM8** – Plasmid generated by PCR amplification of Truncated sequence (1 – 609 aa) of CCNA_00136 (*ampG*) using pMM7 as a template with F.P – CCCCATATGATGTCCGAAGAAGCCAAG and R.P. – CCCCGGTACCCAGGCCCACGACCAGACC digested with Nde1 and Kpn1 and ligated into digested vector pXMCS-5.

**pMM9** – Plasmid generated by PCR amplification of CCNA_02650 *(ampD)* from CB15N genomic DNA using primers F.P – CCCCCATATGATGAGCCTGTCCCTGATC and R.P – CCCCGGTACCTCAGTCGGCCGCCGCCCG, digested with Nde1 and Kpn1 and ligated into digested vector pJS14.

**pMM10** – Plasmid generated by PCR amplification of Truncated sequence (1 – 441 aa) of CCNA_02650 (*ampD*) using pMM10 as a template with F.P – CCCCCATATGATGAGCCTGTCCCTGATC and R.P. – CCCCGGTACCCCCGGATCGATCTTGCGG, digested with Nde1 and Kpn1 and ligated into digested vector pXMCS-5.

**pMM11** – Plasmid generated by PCR amplification of CCNA_00136 (*ampG*) using pMM7 as a template with F.P - CCCGGTACCATGTCCGAAGAAGCCAAG and R.P – CCCCGCTAGCTTAGGAGGCGGCGAGGGT, digested with Kpn1 and Nhe1 and ligated into digested vector pXGFPN-2.

**pMM12 and pMM14 –** Plasmid generated by PCR amplification of truncated sequence (1-300 aa) of CCNA_01945 (*anmK*) from CB15N genomic DNA using primers F.P – CCCCATATGATGACCGGCACCTCGCTG and R.P – CCCCGGTACCGGAAGTCGAACGCCACCG, digested with Nde1 and Kpn1 and ligated into digested pMCS-1 and pXMCS-5 vectors.

**pMM13 and pMM15** - Plasmid generated by PCR amplification of truncated sequence (1-380 aa) of CCNA_03649 (*amgK*) from CB15N genomic DNA using primers F.P – CCCCATATGGTGACCTTGAGTTCTGAA and R.P – CCCCGGTACCGGGCGTAGAGATCATCGC, digested with Nde1 and Kpn1 and ligated into digested pMCS-1 and pXMCS-5 vectors.

**Strain Construction**

Strains RP47 and RP56 were constructed by Homologous recombination method. Plasmids pMM1 and pMM6 were transformed in S17 competent cells, followed by conjugation with the CB15N strain. Colonies obtained upon conjugation were grown overnight in PYE media and about 6µl were spread on PYE plates supplemented with 5% sucrose. Obtained colonies were simultaneously streaked on PYE kanamycin and PYE streptomycin plates for second crossover selection. Colonies obtained on PYE streptomycin selection were further confirmed by gene-specific forward primer and streptomycin cassette reverse primer.

Strains RP48, RP49, RP50, RP51, RP57, and RP63 overexpressing genes under the control of xylose promoter were constructed by transforming plasmids pMM2, pMM3, pMM4, pMM5, pMM7, and pMM9 respectively in S17 competent cells with chloramphenicol selection and then conjugated with the CB15N strain.

Depletion strains RP64, RP66, and RP67 were constructed by transforming plasmids pMM10, pMM12, and pMM13, respectively in S17 competent cells and selected on Streptomycin cassette resistance. The transformed colonies were then conjugated with the CB15N strain.

Depletion strains RP59, RP68, and RP69 were constructed by transforming plasmids pMM8, pMM14, and pMM15, respectively in S17 competent cells and selected on tetracycline resistance. The transformed colonies were then conjugated with the CB15N strain.

Strains RP52, RP53, RP54, RP55, RP60, RP70, and RP71 were constructed by transduction of strains RP48, RP49, RP50, RP51, RP59, RP68 and RP69 with transducing lysate of strain RP47 followed by selection on PYE streptomycin agar solid media.

Strain RP58, RP72 and RP73 were constructed by performing transduction of strain RP57, RP68, and RP69 with transducing lysate of strain RP56 followed by selection on PYE streptomycin agar solid media.

Strain RP61 was constructed by performing transduction of strain RP48 with transducing lysate of RP2. Transduction of strain RP48 with transducing lysate of RP3 (1) yielded strain RP62.

Strain RP65 was constructed by performing transduction of strain RP63 with transducing lysate of strain RP64 followed by selection on PYE streptomycin agar solid media.

Strain RP74 was constructed by transforming plasmid pMM11 in S17 competent cells and with selection on kanamycin resistance, followed by conjugation with CB15N strain.

**Supplementary figure**


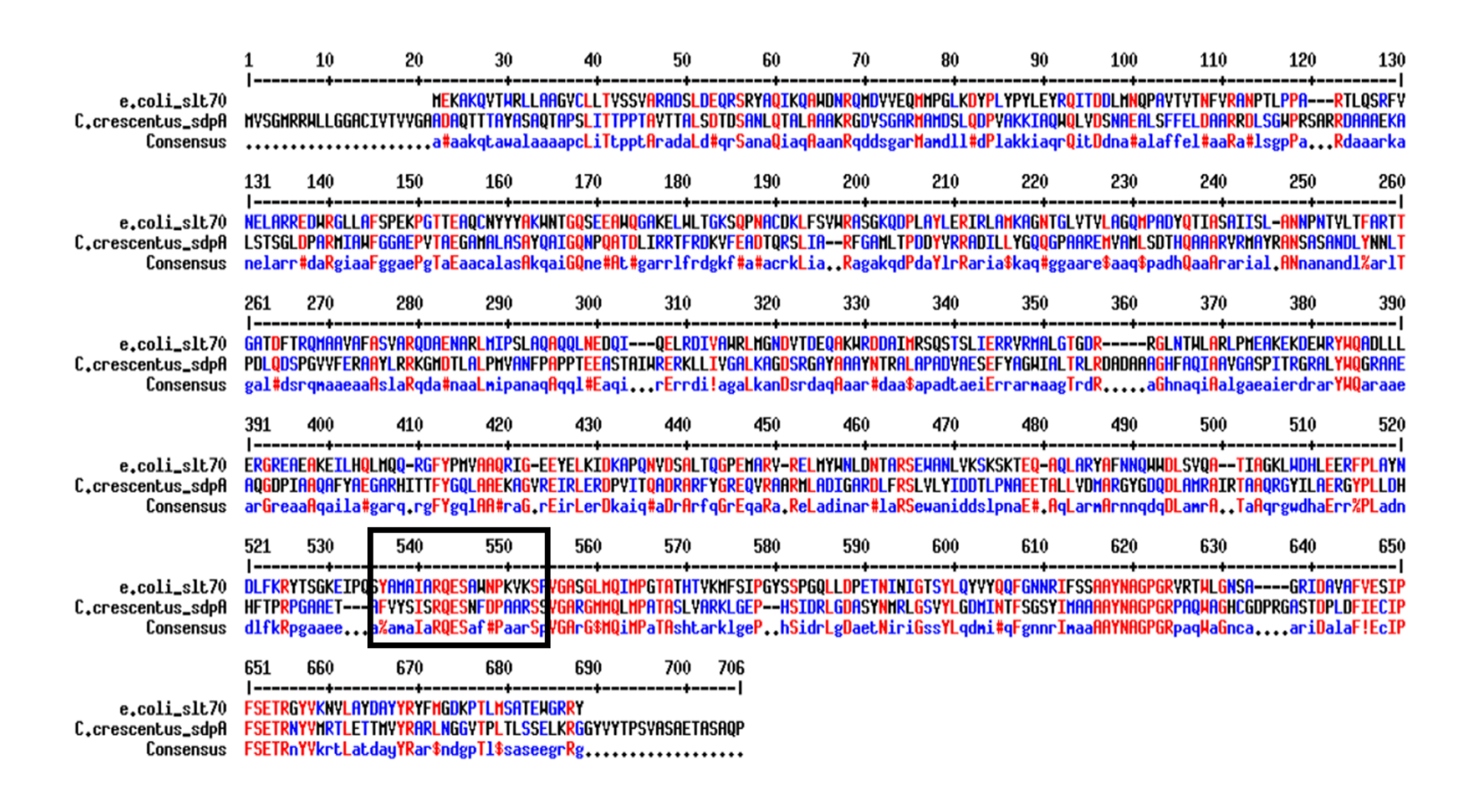


**Supplementary figure 1: Bioinformatics analysis of SdpA of *C. crescentus* and Slt70 of *E. coli***

Multiple Sequence Alignment of *C. crescentus* SdpA sequence and *E. coli* Slt70 in MULTALIN (3). Highly conserved residues are represented by red color, weakly conserved residues by blue, whereas a dot in the consensus line represents no conserved residue. The conserved active site residues are boxed in black.


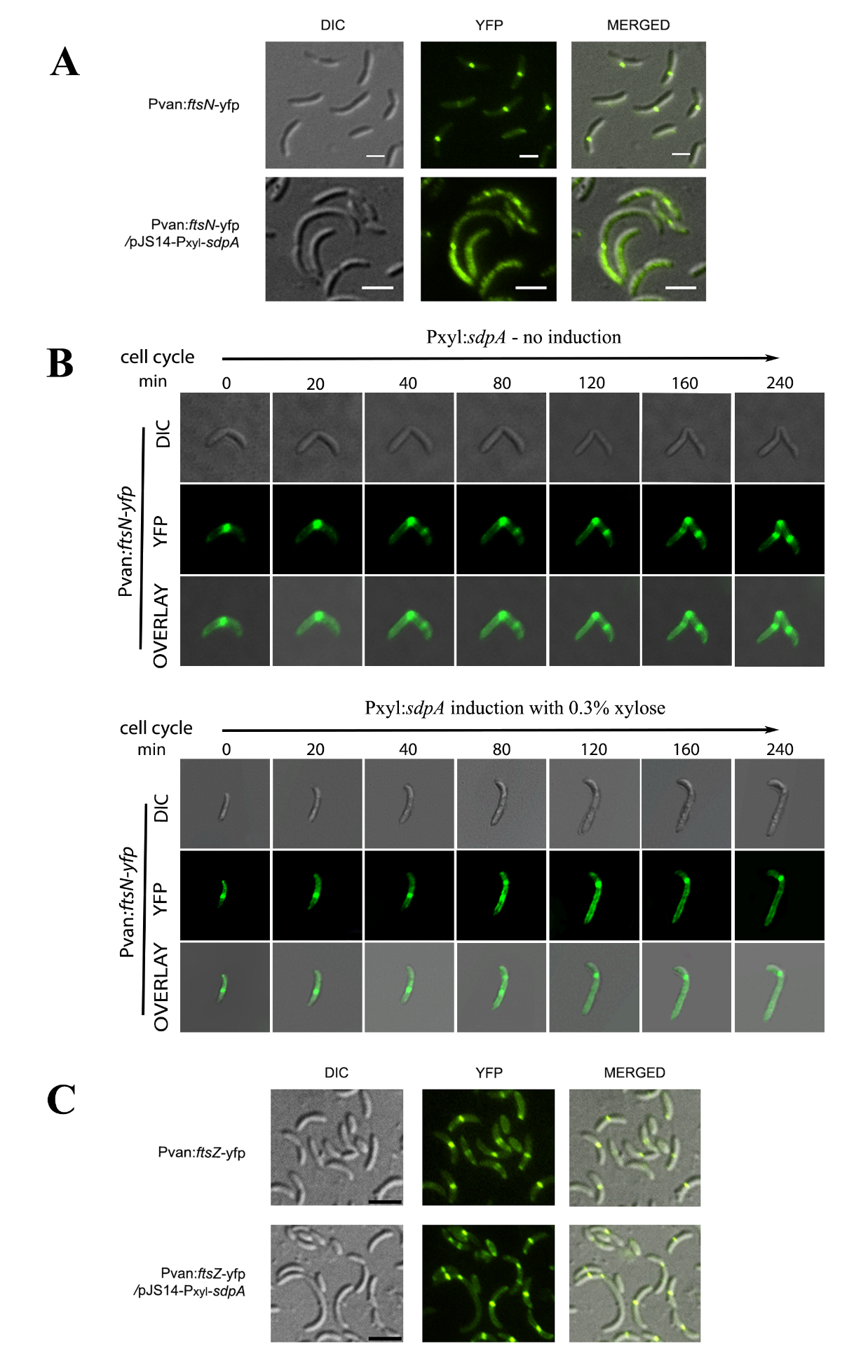


**Supplementary figure 2:**

**(A) SdpA overexpression does not affect mid-cell localization of FtsZ.**

Medial localization of FtsZ remains undisturbed on overexpression of SdpA. Strain RP62 (CB15N Pvan:*ftsZ*-*yfp*/pJS14/Pxyl-*sdpA)* was grown to mid-exponential phase and was induced with 50mM vanillic acid to express *ftsZ-yfp* and 0.3% xylose for overexpression of *sdpA.* DIC and Fluorescence Imaging was performed after 4hrs of induction at 30^°^C.

**(B & C) SdpA overexpression affects the localization pattern of FtsN.**

The localization pattern of FtsN was observed in RP61 (CB15N vanA::Pvan-*ftsN-yfp/*pJS14-Pxyl-*sdpA)* by Confocal Microcopy at 30^0^C. Strain RP62 was grown to mid-exponential phase and was induced with 50mM vanillic acid for expression of FtsN-YFP along with 0.3% xylose for *sdpA* overexpression on PYE agarose pads. Control cells were imaged in the presence of 0.2% glucose. Fig. 2B represents time course imaging performed post-induction with 50mM vanillic acid and 0.3% xylose and Fig.2C represents time-lapse imaging performed on a PYE agarose pad supplemented with 0.5mM vanillic acid and 0.3% xylose.


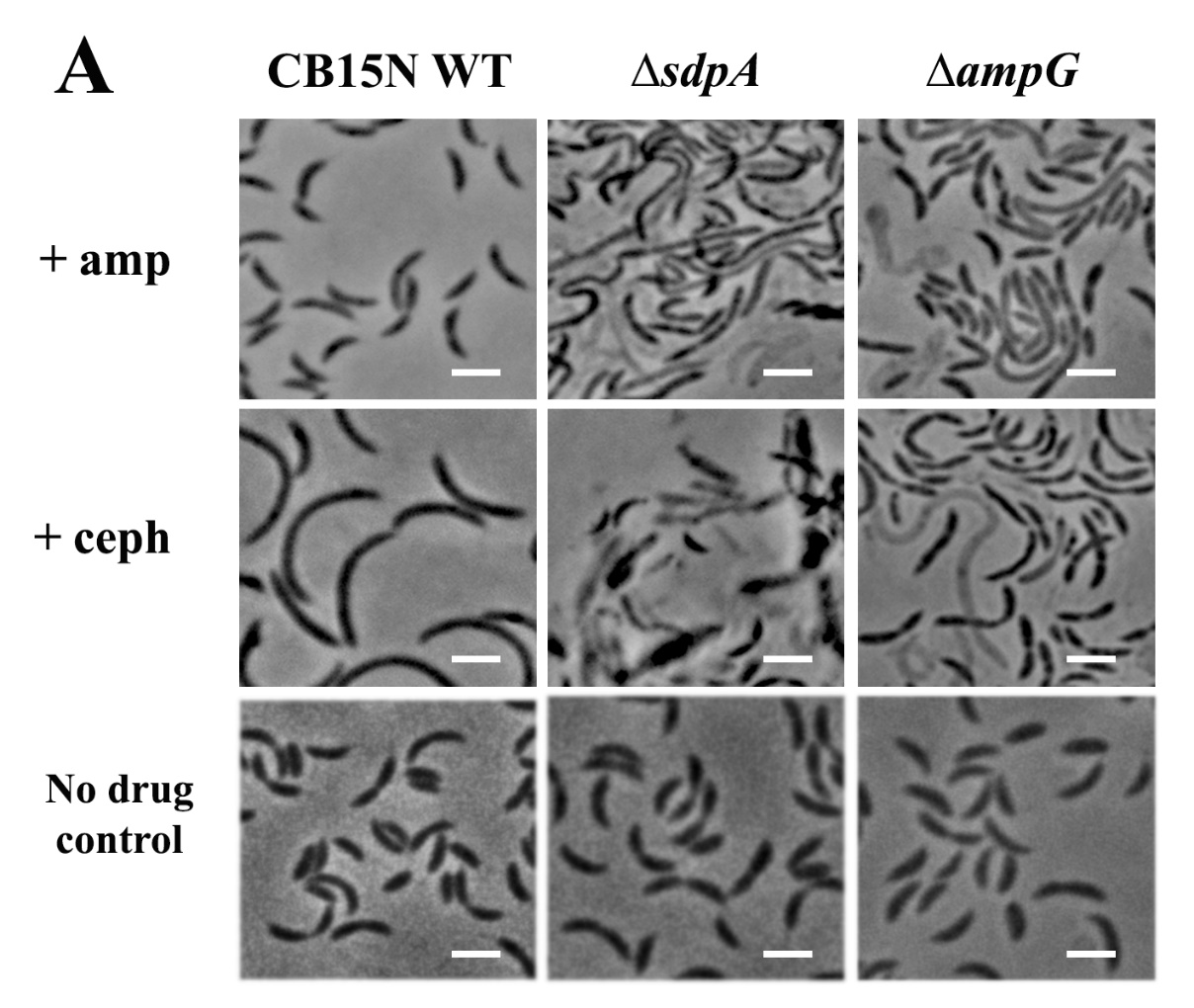


**Supplementary figure 3**: **Morphological defects in PG recycling mutants under β-lactam pressure.**

Phase contrast imaging of strains – CB15N, RP47 (*∆sdpA),* RP56 *(∆ampG)* in the presence of 40µg/ml ampicillin and 8µg/ml cephalexin. The third panel represents no drug control of WT and mutants. Above mentioned strains were treated with β-lactam antibiotics at 0.2O.D_600nm_ and imaged after 6 hrs.


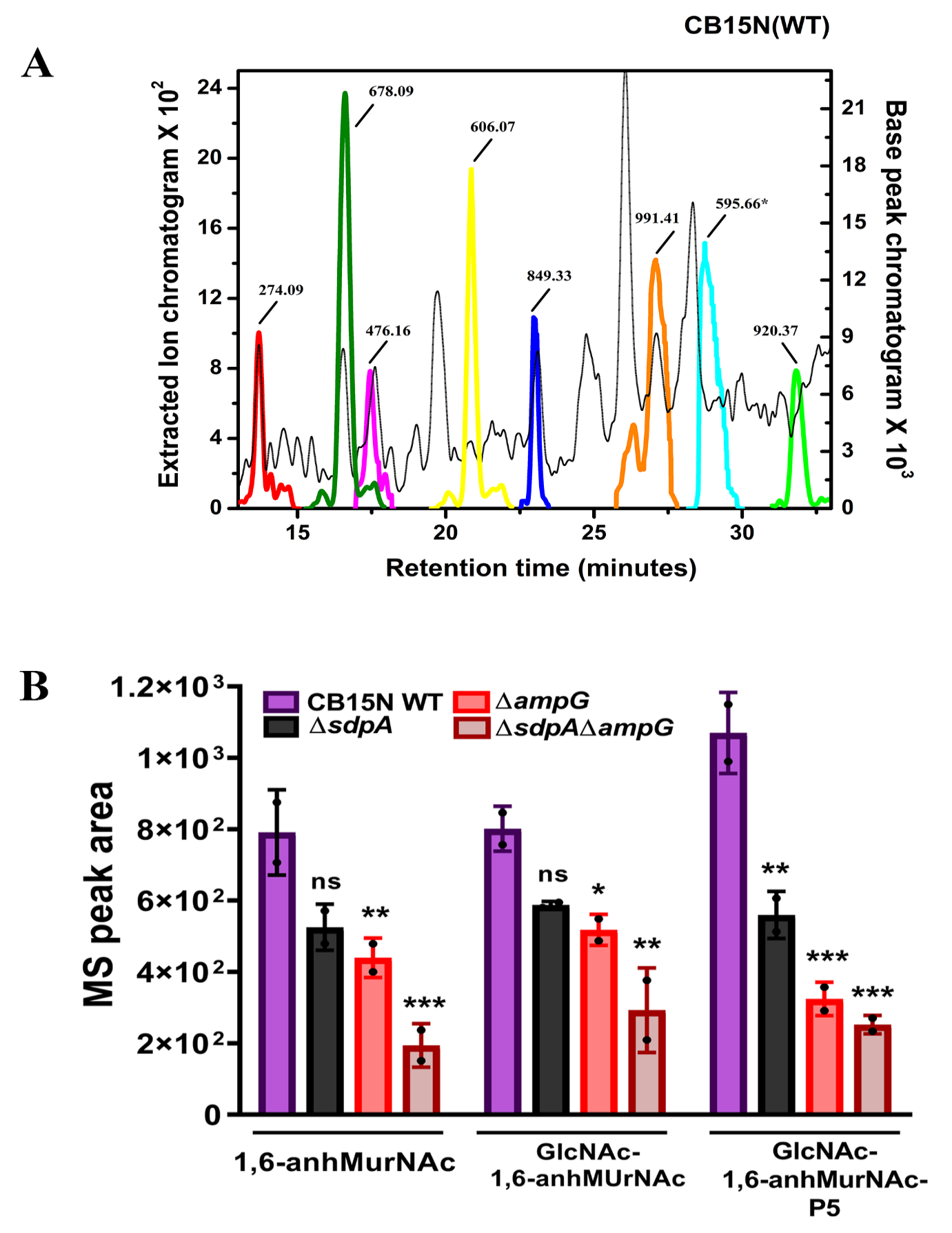


**Supplementary figure 4: LC-MS analysis of soluble muropeptides in PG recycling mutants.**

(A) Base peak chromatogram (BPC) and Extracted ion chromatogram representing various soluble PG fragments that accumulated inside CB15N WT strain detected by LC-MS. Each peak represents m/z values of muropeptides listed in Table No.2 (* = (M-2H)^2-^) .

(B) Graph representing LC-MS quantification of soluble PG muropeptides viz., 1,6-anhMurNAc, GlcNAc-1,6-anhMurNAc and GlcNAc-1,6-anhMurNAc-P5 in strains CB15N, RP47 (∆*sdpA*), RP56 (∆*ampG*), and RP60 (∆*sdpA*∆*ampG*). Mean values of three biological replicates are plotted. Error bars represent standard deviation. (*= p<0.05, **=p<0.005 by T-test vs WT and RP60.

**
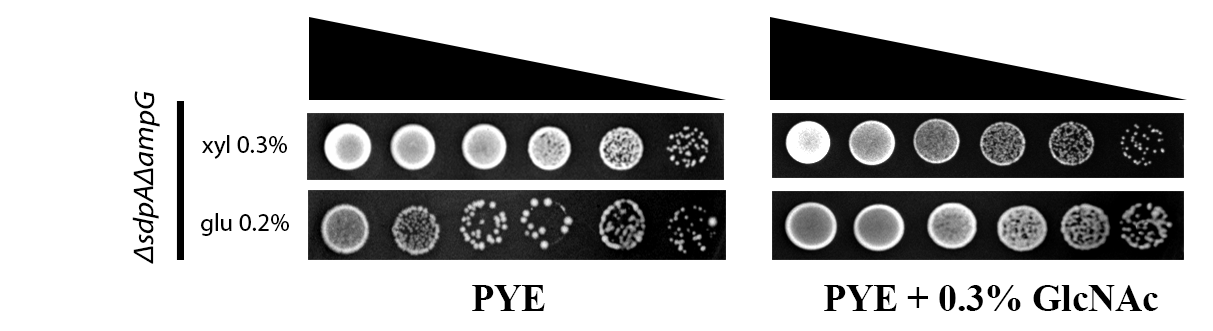
**

**Supplementary figure 5:** Double knockout mutants (∆*sdpA*∆*ampG*) viability was restored to wildtype in the presence of GlcNAc sugar. Serially diluted strain RP60 (*∆sdpA∆ampG)* spotted on medium (PYE + xylose) or lacking inducer (PYE + glucose) supplemented with 0.3% GlcNAc.

| **Schematic** | **Composition** | Ion (M-H)- | | Difference |
| --- | --- | --- | --- | --- |
|  |  | **Expected** | **Observed** | **(ppm)** |
| _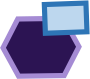_ | 1,6-anhydroMurNAc **(anhM)** | 274.0932 | 274.0940 | 2.91 |
| 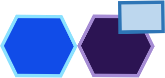 | GlcNAc-1,6-anhydroMurNAc  **(G-anhM)** | 476.1648 | 476.1636 | -2.52 |
| **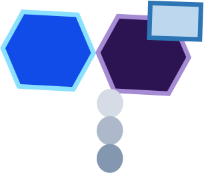** | GlcNAc-1,6-anhydroMurNAc-L-Ala-D-Glu-m-DAP  **(G-anhM-3)** | 849.3371 | 849.3410 | 4.59 |
| **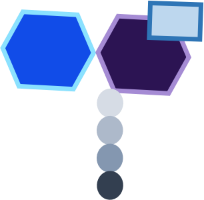** | GlcNAc-1,6-anhydroMurNAc-L-Ala-D-Glu-m-DAP-D-Ala  **(G-anhM-4)** | 920.3742 | 920.3810 | 7.38 |
| **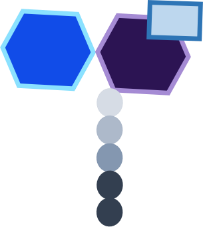** | GlcNAc-1,6-anhydroMurNAc-L-Ala-D-Glu-m-DAP-D-Ala-D-Ala  **(G-anhM-5)** | 991.4113 | 991.4154 | 4.13 |
| **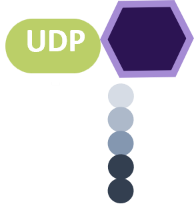** | UDP-MurNAc--L-Ala-D-Glu-m-DAP-D-Ala-D-Ala  **(UDP-M-5)** | 1192.3341  ^*^595.6634 | 1192.3222  *595.6627 | -9.98  -1.17 |
| **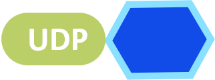** | UDP-GlcNAc | 606.0743 | 606.0727 | -2.63 |
| **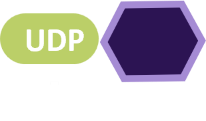** | UDP-MurNAc | 678.0954 | 678.0929 | -3.68 |

**Supplementary Table 1-** List of identified muropeptides in LC-MS, where * represents (M-2H)^2-^ m/z muropeptide.

| **Schematic** | **Composition** | Ion (M+2H)^2+^ | | Difference |
| --- | --- | --- | --- | --- |
|  |  | **Expected** | **Observed** | **(ppm)** |
| **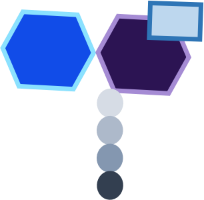** | GlcNAc-1,6-anhydroMurNAc-L-Ala-D-Glu-m-DAP-D-Ala  **(G-anhM-4)** | 461.6980 | 461.6973 | -1.51 |
| **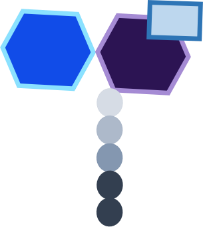** | GlcNAc-1,6-anhydroMurNAc-L-Ala-D-Glu-m-DAP-D-Ala-D-Ala  **(G-anhM-5)** | 497.2166 | 497.2181 | 3.01 |

**Supplementary Table 2-** List of identified muropeptides in LC-MS, where * represents (M+2H)^2+^ m/z muropeptide in periplasmic muropeptide analysis.

|  | WT | ΔsdpA | ΔampG |
| --- | --- | --- | --- |
|  |  |  |  |
| Monomers | 27.61 | 29.79 | 30.11 |
| Dimers | 43.54 | 44.88 | 44.55 |
| Trimers | 25.66 | *22.75 | *22.86 |
| Tetramers | 3.19 | 2.58 | 2.49 |
|  |  |  |  |
| Dipeptide | 0.19 | 0.28 | 0.11 |
| Tripeptide | 6.99 | 7.79 | 6.84 |
| Tetrapeptide | 70.89 | 69.95 | 70.07 |
| Pentapeptide | 21.93 | 21.98 | 22.98 |
|  |  |  |  |
| Crosslink | 41.27 | 39.54 | 39.38 |
| Anhydro | 13.38 | *10.71 | *11.32 |
| Glycine | 14.69 | 15.88 | 15.52 |

**Supplementary Table.3 -** Summary data of PG features in the strains analysed. * = p<0.05 by T-test vs WT.

**Supplementary Movie 1** – CB15N treated with ampicillin (40µg/ml).

**Supplementary Movie 2** - *∆sdpA* (RP47) treated with ampicillin (40µg/ml).

**Supplementary Movie 3** – CB15N treated with cephalexin (5µg/ml).

**Supplementary Movie 4** - *∆sdpA* (RP47) treated with cephalexin (5µg/ml).

**Supplementary Movie 5** – CB15N treated with mecillinam (15µg/ml).

**Supplementary Movie 6** - *∆sdpA* (RP47) treated with mecillinam (15µg/ml).

References

1. Dubey A, Priyadarshini R. 2018. Amidase activity is essential for medial localization of AmiC in *Caulobacter  crescentus*. Curr Genet 64:661–675.

2. Thanbichler M, Iniesta AA, Shapiro L. 2007. A comprehensive set of plasmids for vanillate- and xylose-inducible gene expression in Caulobacter crescentus. Nucleic Acids Res 35:e137.

3. Corpet F. 1988. Multiple sequence alignment with hierarchical clustering. Nucleic Acids Res 16:10881.
